# Toward Skin-like Sensors: Stretchable Conductive Gels for Triboelectric Applications

**DOI:** 10.3390/gels12020151

**Published:** 2026-02-08

**Authors:** Zejun Shen, Na Li, Jianjing Yi, Xiuru Xu, Xiaoxiao Mo, Ruopeng Wang

**Affiliations:** State Key Laboratory of Radio Frequency Heterogeneous Integration, College of Physics and Optoelectronic Engineering, Shenzhen University, Shenzhen 518060, China

**Keywords:** stretchable conductive gels, triboelectric nanogenerator, skin-like sensors, self-powered sensing, wearable electronics

## Abstract

With the rapid development of artificial intelligence and wearable electronics, there is an increasing demand for skin-like, flexible, and self-powered sensors capable of continuously perceiving mechanical stimuli and human motions. Triboelectric nanogenerator (TENG)-based sensors incorporating stretchable conductive gels represent a promising approach to meet these requirements by combining soft mechanical compliance with efficient electromechanical signal transduction. However, conventional metallic or composite electrodes often suffer from mechanical mismatch with soft skin-like systems, motivating the exploration of intrinsically soft and stretchable conductive gels. In this review, we present a comprehensive and structured overview with comparative perspectives of stretchable skin-like conductive gel-based triboelectric devices. First, different classes of conductive gels, including hydrogels, organogels, ionogels, and other emerging gel systems, are systematically summarized and compared in terms of their composition, crosslinking strategies, conductivity, and mechanical characteristics. Next, the pivotal role of conductive gels in bridging skin-like sensing functions and triboelectric applications is elucidated, highlighting how their intrinsic softness, stretchability, self-healing capability, and interfacial conformability enable intimate skin contact and reliable electromechanical coupling. The key performance attributes of gel-based skin-like triboelectric sensors, including stretchability, self-healing behavior, optical and thermal tolerance, electrical durability, and environmental stability, are critically discussed with representative examples and comparative analysis. Typical device configurations, such as thin-film, fiber-shaped, and textile-based architectures, are further reviewed to illustrate structure–function relationships and application-oriented design strategies. Finally, current challenges, limitations, and future research directions for stretchable conductive gel-based triboelectric systems are outlined, aiming to provide practical guidelines and insights for the rational design of high-performance skin-like triboelectric sensors based on conductive gels.

## 1. Introduction

The skin is the largest organ in the human body, covering an area of 1.5 to 2 m^2^ and accounting for approximately 15% of total body weight [1]. Beyond its role as a protective barrier, human skin possesses remarkable properties that have inspired the development of skin-like electronic devices. The design of these devices seeks to replicate the skin’s exceptional flexibility, stretchability, and sensory capabilities, while also exploring new functionalities. An ideal skin-like electronic device should not only mimic these properties but, in some cases, enhance them. For example, the Young’s modulus of human skin ranges from 100 to 600 kPa, demonstrating its elasticity and ability to deform under various mechanical stresses. These attributes enable the skin to adapt to bending, stretching, and recovery, particularly in areas such as joints. Thus, skin-like devices must be sufficiently flexible and stretchable to replicate the dynamic behavior of skin. Furthermore, the skin’s ability to self-repair when damaged through its natural healing mechanisms highlights the potential for incorporating self-healing materials into skin-like devices [2].

Human skin is also a highly sensitive organ, capable of detecting a wide range of stimuli, including pressure, temperature changes, vibration, and even pain. Receptors such as Meissner corpuscles and Pacinian corpuscles are integral to this sensory function, enabling skin to sense both static and dynamic forces [3]. Skin-like devices, therefore, need to replicate this sensory versatility, allowing them to detect external forces, temperature changes, and even visible light [4]. For practical applications, these devices should be flexible, stretchable, self-healing, and tunable to meet the demands of different sensing environments.

A triboelectric nanogenerator (TENG) is a self-powered device that converts mechanical energy into electrical energy through triboelectric effects and electrostatic induction [5]. TENGs have gained significant attention due to their ability to serve as both energy harvesters and sensors for detecting mechanical motion [6]. Depending on their operating modes, TENGs can be classified into single-electrode, independent triboelectric layer, vertical contact-separation, and lateral sliding modes [7,8,9,10]. Among these, flexible and stretchable TENGs, referred to as skin-like TENG devices, require components, such as conductive electrodes, that are also flexible or stretchable. However, many conventional flexible TENGs still rely on metallic films, carbon-based layers, or polymer composites that exhibit limited stretchability, poor conformability, and susceptibility to interfacial failure under repeated deformation [11]. These drawbacks restrict their long-term reliability and practical applicability in skin-like and wearable systems, thereby motivating the exploration of intrinsically soft and stretchable conductive gels as alternative electrode materials. Nevertheless, compared with conventional rigid or metallic electrode-based TENGs, gel-based triboelectric devices often exhibit relatively lower electrical output and higher susceptibility to performance fluctuation, which remains a critical issue to be addressed.

The role of conductive gels in bridging the concepts of “skin-like sensors” and “triboelectric” applications is pivotal. In a typical gel-based TENG, conductive gels primarily function as stretchable and deformable electrodes, while the triboelectric charge generation mainly occurs at the interface between two triboelectric layers. Mechanical contact and separation induce surface charge transfer, and the resulting electrostatic induction drives electron flow through the conductive gel electrodes to generate electrical signals. Therefore, the dielectric properties of triboelectric layers, together with the electrical conductivity and interfacial conformability of gel electrodes, jointly determine the output performance of gel-based TENGs. Conductive gels are soft, stretchable materials that can maintain electrical conductivity while conforming to the shape of human skin or deformable surfaces. These materials enable the development of devices that are both skin-like in their flexibility and stretchability and capable of functioning in triboelectric systems. The inherent softness and flexibility of conductive gels make them ideal for use in wearable, skin-like devices, where they ensure intimate contact with the skin, thereby improving performance in triboelectric energy harvesting and sensing applications [12]. In contrast to rigid metallic electrodes, which can cause issues such as fatigue failure or detachment from the skin, conductive gels form conformal, flexible interfaces that mitigate these problems, particularly under cyclic deformation, bending, or compression—critical challenges for long-term wearability in real-world conditions. From a broader perspective, this integration establishes a continuous dynamic mechanical information transduction pathway at both material and device levels.

A gel is a non-fluid colloidal or polymer network expanded throughout its volume by a fluid. Conductive gels, composed of a polymer matrix and electrically or ionically conductive components, are key to transmitting electrical signals in skin-like devices. These gels exhibit essential properties such as stretchability, biocompatibility, and tunable conductivity, which are crucial for the development of wearable devices that interact closely with the skin. Moreover, by adjusting their microstructure and material composition, properties such as self-healing, thermal stability, and environmental resilience can be engineered to meet the specific needs of skin-like triboelectric devices. Hydrogels, for example, not only enhance the mechanical properties of these gels but also provide hydration and lubrication at the interface, improving user comfort during wear. Furthermore, self-healing conductive gels address the wear and tear typical in practical wearable applications, offering the durability needed for long-term use.

In conclusion, conductive gels serve as a crucial element in bridging skin-like sensors and triboelectric applications. In this review, we systematically summarize different classes of conductive gels, analyze their key structure–property relationships, and discuss their roles in enabling stretchable, self-healing, and durable skin-like triboelectric devices. In this review, “skin-like” primarily refers to the mechanical softness, stretchability, conformability, damage tolerance, and multifunctional sensory capability of devices, rather than strict biological replication.

## 2. Stretchable Skin-like Conductive Gel Types

**Figure 1:**
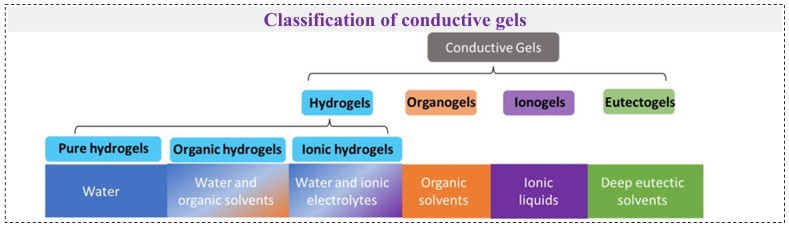
Classification of conductive gels based on their swelling phase or active species.

Balancing electrical or ionic conductivity with mechanical elasticity remains a core challenge in the development of stretchable conductive gels. In this review, we examine engineering strategies for these elastic conductors, organized according to the types of gels used. Given the complexity of gel classification (Figure 1), we primarily categorize stretchable conductive gels into hydrogels (including pure hydrogels and hydrogels with additives, such as organic hydrogels and ionic hydrogels), organogels, and ionogels based on the composition of their liquid phase [13,14]. The performance comparison of various gels is shown in Table 1. In addition, we briefly discuss other emerging types of elastic conductive gels, such as deep eutectic gels and shear stiffening gels. Aerogels, which are typically non-conductive and possess poor stretchability, are excluded from our discussion.

### 2.1. Conductive Hydrogels

Hydrogels are gels with water as their liquid phase [15]. The hydrophilic groups within the polymer chains trap water molecules and form a three-dimensional network structure through cross-linking, resulting in a relatively stable gel system. Additionally, because of the swelling behavior of the hydrophilic groups, hydrogels can absorb a significant amount of water, leading to volume expansion and water retention capacity at room temperature [16]. Hydrogels are usually made using elastic polymers such as poly(vinyl alcohol) (PVA) and poly(acrylamide) (PAAm). These polymers provide stretchability and optical transparency but do not naturally offer conductivity. Hydrogels made of a single polymer network are called homopolymer network hydrogels, while those with additional networks reported through physical or chemical treatment are known as multi-network hydrogels [17].

Without extra additives, homopolymer network hydrogels generally have low stretchability and/or poor conductivity, lack functional tuning, and have limited mechanical performance, which restricts their use in multifunctional skin-like devices. In contrast, multi-network hydrogels, which incorporate various polymers and additives to form multiple network architectures, show improved mechanical strength, better structural stability, and greater functional versatility. Building on either single- or multi-network systems, various electromechanical properties can be tailored by introducing conductive nanomaterials such as conducting polymers (polyaniline (PANi), polypyrrole (PPy), and poly(3,4-ethylenedioxythiophene):poly(styrene sulfonate) (PEDOT:PSS), etc.), carbon-based conductive materials (graphenes, carbon nanotubes (CNTs), carbon nanofibers (CNFs), super carbon black (CB), etc.), metallic nanostructures (silver/copper/gold/platinum nanoparticles, nanowires, nanoplates, etc.), and other conductive materials like two-dimensional materials (like MXene). It is important to note that carbon-based, metal-based, and MXene-based conductive hydrogels can also be developed in the form of organogels and ionogels. Furthermore, organic hydrogels can be formed by partially replacing volatile water molecules with non-volatile organic small molecules. Because of their high water content, hydrogels can also be made ionic (known as ionic hydrogels) by adding electrolytes such as charged polyelectrolytes, zwitterionic species and ions (Li^+^, Na^+^, Fe^3+^, Cu^2+^, Zn^2+^, etc.).

### 2.2. Conductive Organogels

Organogels are gels with organic solvent as their liquid phase [18]. The organic molecules within the matrix interact with the polymer chains and participate in cross-linking to form a three-dimensional network structure. While organogels share some similarities with organic hydrogels formed by partial replacement of water molecules with organic small-molecule solvents, their key distinction is that organic hydrogels retain a certain amount of water molecules, whereas organogels are essentially water-free [19]. Replacing aqueous solvents with organic solvents, or their mixtures, addresses limitations of traditional hydrogels such as poor environmental stability and low mechanical strength. Compared to water molecules in hydrogels, the selection of organic swelling agents for organogels is far broader. For instance, high-boiling-point organic solvents or hydrophobic solvents can be used to significantly enhance the gels’ stability against temperature fluctuation and humidity change. Organic swelling agents play a pivotal role in the performance of organogels. They have been shown to improve functionality under extreme conditions, form specific and strong interactions with the polymer network to enhance flexibility and tensile strength, enable self-healing by facilitating the sliding and reformation of broken bonds, and improve the overall environmental tolerance of the gel [18]. For example, organogels based on dynamic π–π stacking interactions [20] have demonstrated substantial improvement in mechanical strength and stretchability. Although organogels formed by π–π stacking interactions may exhibit limited intrinsic conductivity, this is a general characteristic of organogels and can be addressed by incorporating external conductive fillers. Currently, stretchable conductive organogels are often prepared using solvents such as propylene carbonate (PC) [21], dimethyl sulfoxide (DMSO) [22], and ethylene glycol (EG) [23], which serve both as swelling agents and as chemically stable, flexible media for functional applications.

### 2.3. Conductive Ionogels

Ionogels, also known as ionic liquid gels, use ionic liquids ([EMIM][BF_4_], [EMIM][TFSI], [BMIM][PF_6_], and even a low degree of polymerization poly(ionic liquid), etc.) as their liquid phase [24,25]. These ionic liquids are composed entirely of anions and cations and remain liquid at room temperature. Unlike ionic hydrogels with ionic liquids, ionogels are water molecule-free, and the ions form a three-dimensional network structure through charge interaction, trapping the ionic liquid within the gel and cross-linking to form a stable gel system [26]. Most ionogels have low tensile strength, fracture toughness, and Young’s modulus. However, the crosslinking strength can be adjusted by varying the content of the ionic liquids to polymers, thereby improving the mechanical properties of the ionogels [27]. Ionic conductivity is the most prominent property of ionic liquids. Ionic liquids can impart high electrical conductivity to gels on their own, while hydrogels and organogels typically do not possess electrical conductivity and require the addition of conductive fillers, conductive polymers, etc., to become conductive. This highlights the important role of ionic liquids in stretchable conductive ionogels. In addition, the high initial decomposition temperature of ionic liquids contributes to the high thermal stability of ionogels. The non-volatility and non-flammability of ionic liquids render the environmental stability and safety during operation of the gels under extreme conditions. There is a strong electrostatic attraction between the anions and cations in ionic liquids, which accounts for the high electrochemical stability of ionogels as well. Currently, common cations used in ionogels include imidazolium, pyridinium, piperidinium, and pyrrolidinium, among which imidazolium and pyridinium are frequently used in the preparation of stretchable conductive gels due to their good ionic conductivity and tunable rheological properties. The most commonly used ionic liquid anions in stretchable conductive ionogels are dihydroamine, chloride, and trifluoromethylsulfonimidyl ions.

### 2.4. Other Types of Conductive Gels

Deep eutectic gels are synthesized using deep eutectic solvents (DESs). DES is a homogeneous and transparent liquid formed by gradually mixing two or more components—typically a hydrogen bond acceptor and a hydrogen bond donor—in a specific molar ratio at a defined temperature [28,29]. DESs are known for their good electrical conductivity, high ambient-temperature stability, low toxicity, and ease of preparation. Due to their physicochemical similarity to ionic liquids—while avoiding some of their limitations—DESs are regarded as promising alternatives to conventional ionic liquids. Additionally, DESs are sometimes utilized as plasticizers in ionogels. In recent years, DESs have also been used as gel electrolytes. However, deep eutectic gels often suffer from low conductivity, high hygroscopicity, and poor mechanical properties [30]. Previously, both deep eutectic gels and ionogels were classified as polymeric ionomers [31].

Shear stiffening gels (SSGs) are a distinct subclass of viscoelastic, stretchable conductive gels [32]. These solvent-free gels exhibit a remarkable increase in mechanical strength when subjected to high external strain or shear forces [33]. In addition to their strain-responsive stiffening behavior, SSGs generally possess good impact resistance, making them particularly suitable for wearable device applications—an advantage not typically observed in other gel types.

Plasticizer-based gels are also used in the fabrication of TENGs, with plasticizer-modified poly(vinyl chloride) (PVC) gels being among the most common ones. The incorporation of a proper amount of plasticizer can not only enhance the conductivity of the gel but also improve its stretchability. Moreover, certain plasticizers can further improve optical transparency and frost resistance [34], enabling the development of multifunctional, high-performance TENGs [35].

To provide a comparative overview of representative conductive gels reported for triboelectric applications, Table 1 summarizes typical hydrogel-, organogel-, ionogel-, and deep eutectic gel-based systems in terms of gel type, crosslinking strategy, solvent/ionic phase, electrical conductivity, and mechanical properties. As can be seen, conductive gels exhibit a broad range of conductivities (from ~10^−4^ to >10 S/m) and stretchability (several hundred to thousands of percent strain), reflecting the strong dependence of performance on polymer chemistry, network architecture, and incorporated conductive species. In general, ionogels and deep eutectic gels tend to offer superior environmental stability, while hydrogels often display higher stretchability and softness. Organogels provide a useful compromise between mechanical compliance and solvent retention. These comparisons establish a materials-level basis for the subsequent discussion of how gel properties influence triboelectric device performance.

**Table 1:** Comparison of representative conductive gels for triboelectric applications.

Materials	Types	Crosslinking	Ionic/Solvent Phase	Conductivity (S/m)	Mechanical Properties (Stress/Strain)	Refs.
PC/NaClO_4_/ACMO	Organogel	Single network	PC/NaClO_4_	N.A.	0.075 MPa/474%	[21]
WPU/VMT/Ag emulsion	Deep eutectic gel	Single network	ChCl/GL	0.63	35.34 MPa/1370%	[36]
NH_2_-HBP/AA/Fe^3+^	Ionogel	Single network	[C2mim][EtSO_4_]	0.53	0.043 MPa/812%	[37]
PVDF-co-HFP	Ionogel	Single network	[EMIM][TFSI]	0.0005	0.120 MPa/600%	[38]
PVP/P(AAm-co-[VBIM]Br)/CNF-BA	Ionogel	Dual networks	P(AAm-co-[VBIM]Br)	1.37	0.148 MPa/1750%	[39]
PAAm/PVP	Hydrogel	Single network	H_2_O	0.07	0.85 MPa/2800%	[40]
CA/AC/NVP/LiCl	Hydrogel	Single network	EG/H_2_O	0.8	0.018 MPa/132%	[41]
PAM/sodium alginate/LiCl	Hydrogel	Single network	H_2_O	1.29	0.18 MPa/2100%	[42]
PVA/AMPS/CS/PA	Hydrogel	Dual network	H_2_O/PA	9.4	0.175 MPa/420%	[43]
BWT/Ag^+^/gelatin/borax	Organogel	Single network	PG/H_2_O	14.2	0.045 MPa/530%	[44]
ZnO/AMPS	Ionogel	Dual network	[C2mim][EtSO_4_]	0.31	0.240 MPa/1418%	[45]
SL/GMA	Deep eutectic gel	Single network	LA/ChCl	0.000126 (−40 °C)	1.53 MPa/320%	[46]
PAM/PDA/GO	Hydrogel	Single network	EG/H_2_O	0.0173	N.A./1100%	[47]
PVA/SA/DA/Fe^3+^	Hydrogel	Dual network	H_2_O	0.16	3.14 MPa/442%	[48]
PVA/gelatin/Na_2_B_4_O_7_/CoN/CNT	Organogel	Single network	EG	0.000075	0.145 MPa/530%	[49]
HEMA/MAM/PEG/LiCl	Organogel	Single network	PEG	9.09	0.08 MPa/943%	[50]
DMAPS/[VBIM][BF_4_]	Organogel	Single network	Gly	0.0004	0.08 MPa/6000%	[51]
PVA/PEG 200/Fe^3+^	Organogel	Single network	PEG 200	6.5	8.25 MPa/800%	[52]
PAM/TOCNFs	Ionogel	Dual network	[BMIm]Zn_x_Cl_y_	0.27	5.9 MPa/312%	[53]
PVA/MXene	Deep eutectic gel	Single network	ChCl/Gly	0.17	6.31 MPa/596%	[54]
PAA/Cellulose	Deep eutectic gel	Single network	ZnCl_2_ hydrate /AA/H_2_O	0.072	14 MPa/980%	[55]
IA/ChCl	Deep eutectic gel	Single network	LA/ChCl	0.152	2.2 MPa/540%	[56]

Notes: Data are collected from representative literature. Gel types are classified based on the dominant liquid phase retained in the polymer network; abbreviations are defined in the Abbreviation section; “N.A.” indicates not available.

## 3. Integrating Skin-like Sensors and Triboelectric Applications Through Conductive Gels

### 3.1. Fundamental Roles of Conductive Gels in Skin-like Triboelectric Sensors

The integration of skin-like sensors and triboelectric applications represents an important direction in wearable electronics and self-powered devices. While these two fields are often considered independent, conductive gels serve as the key component that bridges the gap between them, enabling devices that are both flexible and capable of generating energy through triboelectric effects. Skin-like sensors are designed to replicate the properties of human skin, such as flexibility, stretchability, self-healing, and sensitivity to external stimuli. These sensors require materials that can conform to the skin’s surface, stretch without losing performance, and detect a wide range of tactile stimuli, such as pressure, temperature, and vibrations. To make these sensors self-powered, triboelectric systems are employed.

In TENGs, mechanical motion is converted into electrical signals through contact electrification and electrostatic induction. In a single-electrode TENG, one electrode remains stationary while the other is connected to a load, capturing the charge generated by the triboelectric effect. This configuration is especially suitable for applications requiring flexibility and compactness, such as wearable or flexible devices. Conductive gels provide the necessary mechanical and electrical properties to support both functions. Their stretchable nature allows them to conform to the surface of the skin or other deformable substrates, ensuring intimate contact to detect stimuli accurately. At the same time, their conductivity allows them to effectively collect and transmit electrical charge generated during the triboelectric process.

It should be noted that, although stretchable conductive gels enable excellent mechanical compliance and interfacial conformability, their intrinsic ionic conduction, high water content, or polymer-rich composition may limit charge collection efficiency and carrier mobility. As a result, many gel-based TENGs still deliver lower open-circuit voltage and short-circuit current compared with devices employing optimized metallic or carbon-based electrodes. This inherent trade-off between mechanical softness and electrical output remains a central challenge in the design of high-performance gel-based triboelectric systems. By serving as both electrodes in triboelectric systems and sensors in skin-like applications, conductive gels bridge these two domains, enabling the development of self-powered wearable electronics. For example, in wearable healthcare devices, conductive gels can facilitate the real-time monitoring of body movement or environmental changes (via skin-like sensors) while also harvesting energy through triboelectric effects to power the device. Such integration reduces reliance on external power sources such as batteries and supports long-term, autonomous operation of wearable electronics.

### 3.2. Key Properties of Conductive Gels for Skin-like Triboelectric Performance

The performance of triboelectric devices is influenced by several factors, including the materials’ ability to generate and maintain a charge, the efficiency of energy conversion, and the mechanical properties of the device. Conductive gels possess several key properties that significantly enhance the triboelectric performance of devices in which they are used. These properties are crucial for integrating skin-like sensors and triboelectric systems, enabling the development of highly efficient, self-powered, and flexible wearable devices.

Mechanical Properties: One of the most critical properties of conductive gels is their stretchability and flexibility, which allow the gels to conform to curved and irregular surfaces, such as human skin. These properties ensure that the gels maintain their functionality under deformation, such as stretching, bending, and compression. In triboelectric applications, flexibility is essential to maintain consistent contact and efficient charge transfer during mechanical deformation, which is particularly crucial for skin-like triboelectric sensors. The gels must remain functional even when the skin is stretched or bent during movement.

Self-Healing: Many conductive gels exhibit self-healing properties, which are particularly beneficial for wearable devices exposed to wear and tear. These gels can recover from damage caused by mechanical stress, such as stretching or impact, maintaining their electrical conductivity and mechanical integrity over time. The ability to self-heal is critical for ensuring the long-term stability of skin-like sensors and triboelectric devices, which are often subjected to repetitive deformation. Self-healing gels reduce the need for frequent repairs or replacements, thereby improving operational reliability during long-term use.

Optical and Multifunctional Properties: In addition to their mechanical and electrical properties, conductive gels can also exhibit optical and multifunctional capabilities. These properties make them suitable for applications beyond traditional energy harvesting, such as in optical sensing or smart wearable electronics. For instance, conductive gels can be engineered to respond to external light stimuli, changing their optical characteristics such as color or transmittance, enabling light-responsive devices. Such gels can be used in optical triboelectric sensors for applications where both energy harvesting and light sensing are required. Furthermore, multifunctional gels can integrate additional features like temperature sensing or pressure detection, expanding their use beyond energy generation. These gels enable the development of hybrid devices that combine multiple sensing and energy-harvesting functionalities in a single, flexible platform. This makes them ideal for applications in smart textiles, biomedical sensors, and interactive wearable devices, where multiple types of stimuli need to be detected and acted upon simultaneously.

Environmental Stability: The environmental stability of conductive gels is essential to ensure the longevity and reliability of triboelectric devices. Gels must maintain their electrical properties and mechanical strength when exposed to varying environmental conditions, such as temperature fluctuations, humidity, and UV light. Many conductive gels are designed to remain stable under harsh conditions, ensuring that skin-like triboelectric devices can operate efficiently in environments where moisture, temperature changes, or prolonged exposure to the sun might otherwise degrade performance.

Durability: The durability of gel-based triboelectric sensors is critical for their long-term performance. This includes their ability to withstand repeated deformation and environmental stress without compromising functionality. Key aspects of durability include cyclic stability, interface integrity, and resistance to drying and moisture absorption. Techniques like crosslinking, self-healing, and hydrophobic modification help ensure that the gel-based sensors maintain their electrical conductivity and mechanical flexibility over time, even under harsh environmental conditions.

Overall, the combination of stretchability, conductivity, self-healing capability, environmental stability, and durability makes conductive gels a versatile material platform for uniting skin-like sensing and triboelectric energy harvesting. These attributes underpin their growing use in wearable and self-powered electronic systems, where mechanical comfort and functional integration are as important as electrical performance.

## 4. Properties of Skin-like Triboelectric Sensors Based on Conductive Gels

### 4.1. Stretchability of Gel-Based Triboelectric Sensors

**Figure 2:**
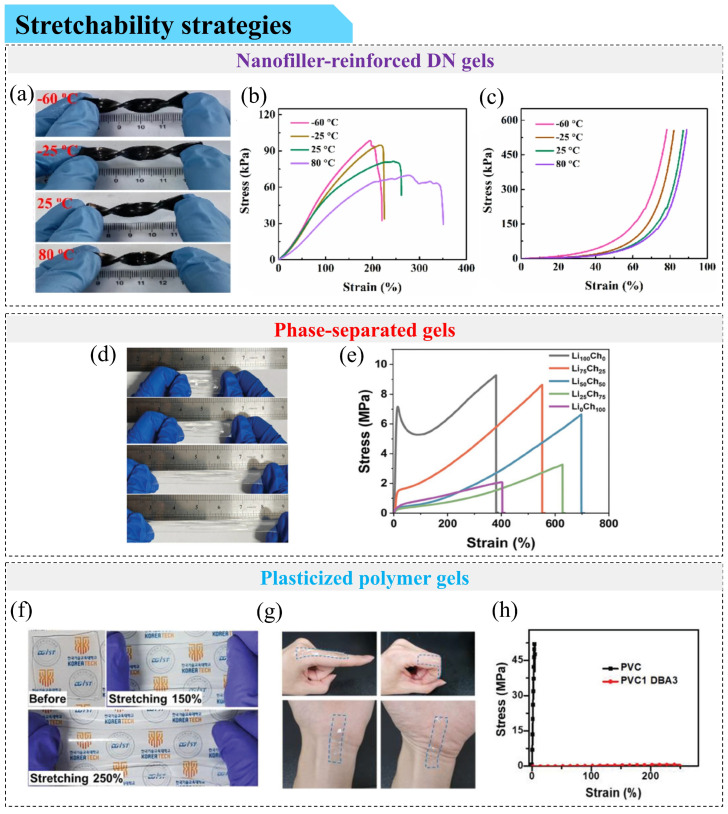
Strategies for achieving highly stretchable conductive gels. (**a**–**c**) Nanofiller-reinforced DN ionic gels: (**a**) Photographs showing the temperature resistance behavior of ionic hydrogel. (**b**) Tensile stress–strain curves of ionic hydrogel at different temperatures. (**c**) Compressive stress–strain curves of ionic hydrogel at different temperatures [58]. Copyright © 2024 Springer Nature. (**d**,**e**) Phase-separated dual-ion-channel eutectic gels: (**d**) Image of a triboelectric sensor based on PSDIC gel stretched to three times its original length. (**e**) Tensile stress–strain curves of PSDIC gels prepared with different lithium salt contents [61]. Copyright © 2025 John Wiley & Sons, Inc. or related companies. (**f**–**h**) Plasticized PVC gels: (**f**) Image of plasticized PVC gel being stretched. (**g**) Images of plasticized PVC gel applied to fingers and wrists. (**h**) Tensile stress–strain curves of plasticized PVC gel [35]. Copyright © 2022 John Wiley & Sons, Inc. or related companies.

The mechanical properties of skin-like triboelectric sensors play an important role in determining their performance and practical applicability. These properties are largely governed by the mechanical characteristics of the constituent gels (Figure 2). An intricate balance of elasticity, strength, and modulus needs to be achieved in gel materials to ensure that the resulting device is robust enough to be handled while remaining soft and compliant for operation—ideally with a modulus approaching that of human skin. A common strategy for achieving the desired mechanical properties is the incorporation of double-network (DN) or multi-network structures, which facilitate energy dissipation. In such systems, a weaker network preferentially fractures under external stress to dissipate energy, while a stronger network preserves the overall structural integrity during handling and repeated operation. For example, Luo et al. developed a fully physically crosslinked DN hydrogel using borax-crosslinked PVA (PVA-B) as the first network and Fe^3+^-crosslinked poly(acrylamide-co-acrylic acid) (P(AM-co-AA)-Fe^3+^) as the second [57]. This hydrogel exhibited a tensile strain of 590%, a tensile stress of 2.1 MPa, and an elastic modulus of 400 kPa—similar to that of the human skin, demonstrating the mechanical benefits of DN architectures. A triboelectric sensor fabricated using this hydrogel as the electrode layer and Ecoflex (a type of soft and stretchable platinum-catalyzed silicone rubber) and polyimide (PI) as friction layers achieved a tensile strain of 160% and a maximum power density of 0.27 W/m^2^, demonstrating satisfactory mechanical and electrical performance.

Beyond network-architecture design, the incorporation of nanofillers as reinforcing or crosslinking elements provides an additional route to improve gel mechanics. Such fillers can increase crosslinking density or introduce sacrificial bond networks to improve elasticity and toughness. Zhao et al. [58] utilized freeze–thaw and ionizing radiation techniques to prepare a DN ionic hydrogel based on poly(ionic liquid)/MXene/PVA (Figure 2a). Two-dimensional Ti_3_C_2_Tx MXene nanosheets served as physical crosslinkers, forming non-covalent interactions with the polymer network. The freeze–thaw cycle facilitated in situ crystallization of PVA for physical crosslinking, while ionizing radiation induced chemical crosslinking of the multi-ionic liquid/PVA network. The resulting ionic hydrogel obtained favorable mechanical performance with tensile stress of 98.6 kPa at 195% strain and compressive stress of 559.4 kPa at 78.3% strainat −60 °C (Figure 2b,c). A single-electrode-mode TENG using this ionic hydrogel as the current collector and Ecoflex as the positive friction layer achieved an open-circuit voltage of 66 V and maintained stable output over 10,000 contact-separation cycles, underscoring its durability under extreme conditions. However, conventional nanofillers often suffer from random dispersion, limiting their reinforcing effect. To address this, Chen et al. [36] developed a hybrid eutectic (DWV) gel with tunable mechanical properties by incorporating silver nanoparticle-loaded two-dimensional vermiculite (VMT) nanosheets as additional physical crosslinking agents. These were combined with waterborne polyurethane (WPU) and a deep eutectic solvent (DES) composed of glycerol (Gly) and choline chloride (ChCl). WPU served as the chemically crosslinked backbone, while VMT nanosheets and DES introduced physical crosslinking. By aligning VMT nanosheets along the tensile direction using an external magnetic field, the gel achieved a strain of 1613% and a tensile stress of 17.22 MPa. A single-electrode-mode TENG using this DWV gel and PDMS as the friction layer exhibited a power density of 0.34 W/m^2^, with stable voltage output over 120 days and consistent electrical performance after 20,000 cycles.

Induced phase separation represents another effective strategy for constructing mechanically robust gel architectures [59]. Similar to multi-network hydrogels, interlocking architectures consist of rigid domains that dissipate energy under stress and flexible domains that reduce stress concentration and accommodate large deformation [60]. Deep eutectic gels, known for their superior ionic conductivity, often suffer from limited mechanical performance. To overcome this, Ma et al. [61] developed a phase-separated dual ionic channel (PSDIC) eutectic gel by blending hydrophilic and hydrophobic DESs and inducing phase separation via in situ polymerization. A triboelectric sensor incorporating the PSDIC gel as the stretchable electrode and VHB/PDMS as the friction layers exhibited 300% stretchability (Figure 2d). The hydrophilic PAA-rich hard phase, containing lithium-ion channels, formed extensive hydrogen bonds and ion-dipole interactions that improved strength. Meanwhile, the hydrophobic poly(hexafluorobutyl acrylate) soft phase, with cholinium cation channels, enhanced toughness. Therefore, this gel achieved a tensile strain of 683.3% and a maximum stress of 6.03 MPa (Figure 2e).

Plasticized PVC gels show favorable mechanical properties for triboelectric sensors as well. Park et al. [35] prepared plasticized PVC gels using dibutyl adipate (DBA) as the plasticizer. Figure 2f shows the images of the plasticized PVC gel during stretching. When applied to the fingers and wrists, it can undergo bending and stretching (Figure 2g). In addition, Figure 2h shows that it has a tensile stress of 1 MPa and a strain of 250%. With the addition of more plasticizers, the elongation at break increases, and tensile strength and modulus decrease [62]. This is due to increased free volume and disrupted chain packing from the plasticizer [63], leading to a physically crosslinked amorphous network. Plasticizer–PVC interactions primarily occur via hydrogen bonding between the plasticizer’s carbonyl (C=O) groups and PVC’s α-hydrogens [64].

Woo et al. [65] studied this effect using three plasticizers—butyl benzoate (BB) (1 carbonyl), dibutyl phthalate (DBP) (2 carbonyls), and tributyl trimellitate (TBT) (3 carbonyls)—and found that elongation at break exceeded 1714% in all gels. As the number of carbonyl groups increased, tensile strength also increased (2.81, 3.74, and 5.47 MPa, respectively), while elongation at break decreased due to steric hindrance, which restricted chain mobility. A BB-based sensor achieved the highest power density of 4.986 W/m^2^—2.1 times and 3.7 times higher than the DBP- and TBT-based sensors—and maintained 50% strain and stable performance over 16,000 cycles at 3 N and 4 Hz. Yang et al. [66] developed a tough PVA-based hydrogel (abbreviated as GMS-PVA hydrogel) with a tensile strain of 1000%, tensile stress of 6.47 MPa, and toughness of 35.24 MJ/m^3^, using a three-step process involving grafting with 3,4-dihydroxy benzaldehyde, metal complexation using FeCl_3_, and salting-out using sodium citrate. Its mechanical robustness is derived from PVA crystalline domains and reversible non-covalent interactions, including hydrogen bonding, π–π stacking, and Fe^3+^–catechol coordination. A triboelectric sensor encapsulating GMS-PVA with Ecoflex withstood rolling and puncture damage, maintaining stable output after 7000 cycles and over 200 rolling cycles under a 50 kg load.

In summary, these examples illustrate that rational network design, filler engineering, phase-structure control, and plasticization are effective routes to achieving highly stretchable gel electrodes suitable for skin-like triboelectric sensors.

### 4.2. Self-Healing Capabilities of Gel-Based Triboelectric Sensors

In practical wearable and flexible applications, triboelectric sensors are inevitably exposed to mechanical damage such as surface scratches, cuts, and interfacial friction. To reduce maintenance during wear and tear and to extend the operational lifetime of these sensors, it is essential to design devices with tailored self-healing capabilities. Analogous to human skin, which can recover from mechanical injury through autonomous repair processes, self-healing TENGs rely on the reversible dissociation and reformation of dynamic covalent bonds or non-covalent interactions within their constituent materials [67].

Ideally, both the friction layer and the electrode layer should exhibit self-healing properties with comparable healing efficiency. However, these two layers are typically composed of chemically distinct materials, and not all components used in TENG fabrication inherently exhibit self-healing behavior. Consequently, current research has primarily focused on developing self-healing polymers and gels—particularly those assisted by shape-memory effects—to impart restorative capability to TENG components [68,69,70]. With the rapid development of self-healing TENGs, increasing attention has been directed toward understanding and improving the healing kinetics, efficiency, and functional recovery of these systems.

#### 4.2.1. Self-Healing Electrodes

**Figure 3:**
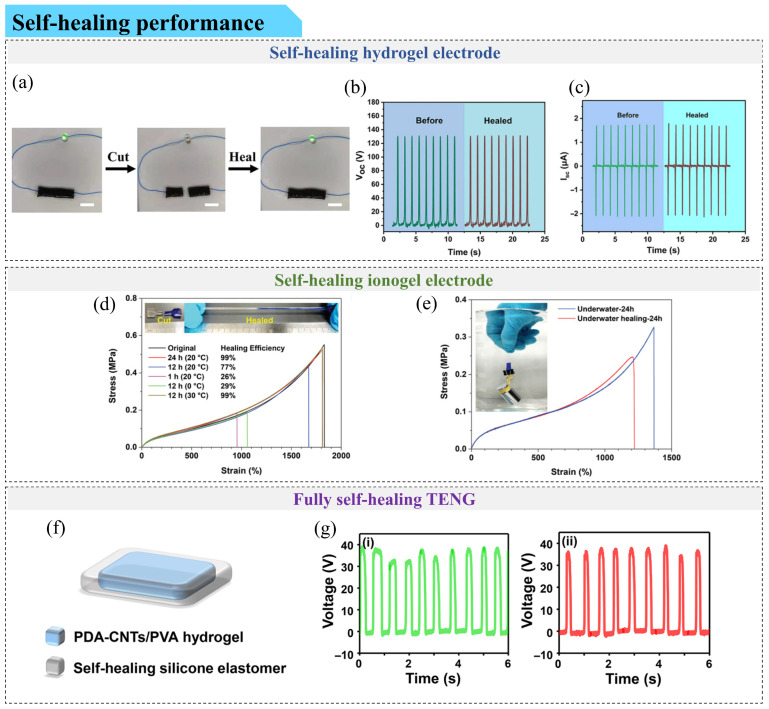
Self-healing performances in gel-based triboelectric sensors. (**a**–**c**) Self-healing hydrogel electrodes: (**a**) Illumination of an LED light after 12 h of self-healing in the organic hydrogel (scale bars: 1 cm). Comparison of (**b**) open-circuit voltage and (**c**) short-circuit current of the O-TENG before and after self-healing [72]. Copyright © 2022 John Wiley & Sons, Inc. or related companies. (**d**,**e**) Self-healing ionogel electrodes: (**d**) Stress–strain curves of original and healed ionogel samples at different healing times and temperatures. (**e**) Underwater stress–strain curves of original and healed ionogel samples after 24 h at room temperature [75]. Copyright © 2021 John Wiley & Sons, Inc. or related companies. (**f**,**g**) Fully self-healing TENGs: (**f**) Schematic structure of the MF-TENG. (**g**) Open-circuit voltage of the original (**i**) and self-healed (**ii**) MF-TENG [83]. Copyright © 2021 American Chemical Society.

Scientists have attempted to develop self-healing electrode layers by embedding silver nanowires or other conductive fillers into self-healing polymers. However, the flexibility of these electrodes is limited, as the conductive filler network tends to break under high strain. In this context, conductive gels have emerged as particularly attractive electrode materials owing to their intrinsic softness, high deformability, and dynamic bonding networks (Figure 3).

Traditional hydrogels, such as those based on PAAm and PVA, exhibit a degree of self-healing capability. When their networks are damaged by external forces, the internal molecules can reform through strong and reversible interactions, such as hydrogen bonding [71]. Zhang et al. [72] loaded silver nanoparticles into reduced graphene oxide and embedded the composite into a PVA-PAM double-network hydrogel. After partially replacing water solvent with Gly, they successfully fabricated an organic hydrogel, which was later combined with silicone rubber to construct a TENG (O-TENG) device. The organic hydrogel self-healed at 25 °C within 12 h, achieving a mechanical self-healing efficiency of 72.5%, and the healed hydrogel restored the ability to illuminate LED lights (Figure 3a). Moreover, the open-circuit voltage and short-circuit current of the O-TENG remained almost unchanged after healing (Figure 3b,c). Wu et al. [73] fabricated gelatin-based hydrogels using gelatin and sodium chloride, which were then immersed in a Gly/aqueous sodium citrate solution for partial solvent replacement and formed organic hydrogels (GNOHs). GNOH demonstrated self-healing at 30–35 °C within 15 min, can easily light up an LED, and achieves a 90% self-healing efficiency. A TENG (AG-TENG) can be subsequently developed using silicone rubber as the friction layer and GNOH as the electrode layer.

Despite these advances, hydrogels and organic hydrogels generally suffer from limited environmental stability. They are vulnerable to extreme temperatures, which can lead to solvent evaporation or condensation, causing reduced ionic conductivity, mechanical flexibility, and poor compression resistance. In contrast, ionogels maintain high ionic conductivity and flexibility across a wide temperature range and are less prone to long-term degradation. Self-healing ionogels generally fall into two types [74]: (1) ionogels crosslinked via non-covalent interactions, in which linear polymers dispersed in ionic liquids form hydrogen bonds, metal-coordination bonds, or ion-dipole interactions; and (2) ionogels crosslinked via dynamic covalent bonds, where chemically crosslinked polymers are embedded in ionic liquids. The latter is relatively rare. Xu et al. [75] synthesized ionogels via a one-pot photoinitiation copolymerization of fluorinated monomer 2,2,2-trifluoroacrylate ethyl (TFEA) and acrylamide (AAm) within a hydrophobic imidazole-based ionic liquid. As shown in Figure 3d, the ionogel healed at 30 °C over 12 h with 99% healing efficiency. Moreover, the ionogel can even heal underwater; after immersion in water at 20 °C for 24 h, 85% healing efficiency was achieved (Figure 3e). However, most self-healing ionogels are limited by mechanical strength, and ionic liquid can extrude from the gel under large mechanical deformation. To mitigate this, strategies such as constructing conductive nanochannels or using ionic liquids that form hydrogen bonds with polymer chains have been explored [76]. Recently, Zhan et al. [77] developed self-healing, stretchable ionogels composed of [EMIM][TFSI] and poly(vinylidene fluoride-co-hexafluoropropylene) (PVDF-co-HFP) containing 88 mol% vinylidene fluoride. The system leveraged both strong polymer crystallization and the weak ionic-dipole interactions. Following heat treatment at 120 °C for 2 min, the fractured ionogels achieved rapid healing, with tensile strength and elongation at break reaching 1.32 MPa and 790% (about half of the pristine values). Nevertheless, the requirement of elevated healing temperature restricts practical applicability.

Deep eutectic gels also show PROMISE for self-healing electrodes. Yang et al. [78] synthesized poly(N-hydroxyethyl acrylamide)/lithium chloride/ethylene glycol (PHEA/LiCl/EG) deep eutectic gels via hydrogen bond self-crosslinking and DES solvent substitution. These gels achieved a tensile strength of 0.68 MPa and can be stretched more than three times the original length, exhibiting robust mechanical performance. After healing at 60 °C for 24 h, they recovered approximately 97% of their original electrical conductivity. When combined with a rubber friction layer, the TENG made from these deep eutectic gels retained handwriting recognition functionality even after 12 days of storage following mechanical damage, confirming their self-healing capability. In addition, the gel exhibits different mechanical healing efficiencies at temperatures of 50–80 °C.

#### 4.2.2. Fully Self-Healing Triboelectric Sensors

When both the friction layer and the electrode layer possess self-healing capabilities, device-level fully self-healing TENGs can in principle be realized. However, in practice, these two functional layers are often based on chemically distinct materials, making it challenging to achieve synchronized healing behavior in terms of time, temperature, and efficiency.

One strategy is the integration of two independently self-healing but chemically dissimilar layers. For example, Li et al. [79] fabricated a fully self-healing TENG (SS-TENG) using a Gly-hydroxyethyl cellulose-based elastomer as the friction layer and a polyacrylamide–carrageenan double-network hydrogel as the electrode layer. Although minor electrical degradation was observed after healing, a pronounced mismatch in healing conditions was found, with the elastomer healing at room temperature within 8 min, whereas the hydrogel required 95 °C for 30 min. A similar limitation was reported by Khan et al. [80], who developed a fully gel-based self-healing TENG (FSASG-TENG) using a conductive poly(lipoic acid)-based gel electrode and a PDMS-based self-healing friction layer. Despite stable output after multiple cutting–healing cycles, the electrode healed within minutes, whereas the friction layer required 24 h, highlighting a notable kinetic incompatibility between the two layers.

To alleviate this issue, another strategy focuses on constructing chemically compatible or unified polymer systems. Joo et al. [81] incorporated an imidazolium ionic liquid diol into a polycaprolactone-based polyurethane to form a self-healable ionic polyurethane (IPU), which served as both a friction layer and an electrode (with Mg powder). The resulting biodegradable TENG reported ~90% voltage recovery and a power density of 436.8 mW/m^2^ after healing. Xu et al. [82] combined a healable shape-memory polymer with a self-healing PVA/NaCl ionic hydrogel to fabricate an HSP-TENG, which recovered its original electrical output after healing at 80 °C.

Beyond material chemistry, structural integration represents an alternative route. Yang et al. [83] constructed a sandwich-type MF-TENG by placing a self-healing PDA–CNT/PVA hydrogel electrode between two self-healing silicone elastomer friction layers, reporting nearly 100% healing efficiency and 150% stretchability after 10 min of healing at 25 °C. Huang et al. [84] further reported an OG-TENG composed of a healable PDMS friction layer and a PAAm-clay organohydrogel electrode, exhibiting reported ultrafast self-healing (~1 s) over −30 to 80 °C and 228% stretchability. Despite these advances, most fully self-healing TENGs still exhibit relatively limited electrical output, largely originating from the modest triboelectric performance of self-healing friction layers. Li et al. [85] partially addressed this challenge by assembling PAA/Zn^2+^ ionogels with self-healing fluorinated polyurethane-urea sheets to fabricate an FSI-TENG, achieving 100% healing efficiency and a high open-circuit voltage of 263 V.

While the representative systems summarized in Table 2 provide a snapshot of current progress, they underscore a broader trend in the field: a transition from fundamental self-healing towards functional restoration under complex stimuli. While a conventional trade-off between energy dissipation (strength) and bond re-association (efficiency) still exists, emerging hierarchical network designs tend to alleviate or partially decouple these parameters, enabling high mechanical robustness (often approaching the MPa level) without necessarily sacrificing rapid healing kinetics. Furthermore, it is noteworthy that for TENG applications, the restoration of electrical conductivity can occur earlier than mechanical healing, a feature that is critical for maintaining sensing continuity but still requires standardized benchmarking across diverse gel platforms.

### 4.3. Optical and Multifunctional Capabilities of Gel-Based Triboelectric Sensors

Human skin exhibits a certain degree of transparency, allowing underlying blood vessels to be visible. It also has a basic sensitivity to light and heat, enabling the perception of sunlight as warmth. In this context, recently developed stretchable conductive gel-based TENGs have demonstrated optical functionalities that extend beyond those of natural skin, enabling multiple modes of visual sensing and optical modulation. Human skin, though translucent, is susceptible to ultraviolet (UV) radiation damage. To address this limitation, Dang et al. [96] developed dual-network ionogels by melting α-lipoic acid (LA) monomer and hydroxypropyl cellulose (HPC), using acrylic acid (AA) as a stabilizer and 1-butyl-3-methylimidazole chloride as a conductive additive. The resulting I-TENG exhibits favorable transparency (84%), satisfactory stretchability of 600%, a wide operating temperature range, self-healing capability, and promising UV resistivity, as evidenced by its almost zero transmittance over the entire UV spectrum (200–400 nm). Beyond UV shielding, conductive gel-based TENGs have also been explored for humidity-responsive optical sensing. In contrast to the limited humidity sensitivity of human skin, Kim et al. [97] developed a TENG incorporating an ionic gel electrode made from 2-hydroxyethyl acrylate, poly(ethylene glycol diacetate), 2-hydroxy-2-methylpropiophenone, and lithium bis(trifluoromethane sulfonyl)imide, and a friction layer made from polystyrene-block-poly(2-vinylpyridine) film interpenetrated with a hydrogel network block copolymer (IHN-BCP). This sensor showed strong humidity sensitivity, with an open-circuit voltage of 3.7 V at 30% RH and 28.6 V at 80% RH in vertical contact separation mode. Moreover, the photonic crystals embedded in the IHN-BCP TENG exhibited structural color changes in response to humidity, enabling real-time visual humidity monitoring. At <30% RH, the film appeared blue or ultraviolet; at 50% RH, it turned green; at 70–80% RH, it shifted to red or infrared. Non-contact sensing was demonstrated with gloved fingers performing vertical and sliding movements. The IHN-BCP TENG was further developed into a self-powered finger motion sensor for visual feedback applications.

In addition to humidity, visualized temperature sensing represents another important optical functionality. Temperature perception is another important biological function of human skin, but it relies on the nervous system and is not directly visible. Wang et al. [98] fabricated thermochromic organic hydrogels (PHOH gels) from PVA and hydroxyethyl cellulose, sodium chloride, water and glycerol (Gly). These gels were incorporated with multiple types of temperature-sensitive particles that reversibly changed color at specific thresholds: blue becomes transparent at 28 °C, red at 35 °C, and green at 45 °C. The resulting PHOP-TENG device enabled multi-level, reversible visual temperature monitoring—despite its limited output voltage. Extending optical functionality into the infrared regime, Wang et al. [99] developed composite hydrogels using acrylamide, agar, sodium tetraborate and tannin-modified black phosphorus (TABP) nanosheets. Integrated with PDMS films bearing micro-pyramidal structures, the resulting Hy-TENG enabled photothermal modulation of temperature via infrared light. The heating response varied with TABP content and irradiation time. Furthermore, the addition of thermochromic inks enabled application in secure communications, achieving dual-layer encryption when two or more thermosensitive inks are added, by tuning the color-change thresholds of multiple inks.

Photoresponsive interfacial behavior has also been incorporated into gel-based systems. Zhang et al. [100] synthesized a light-sensitive adhesive hydrogel from cellulose nanofibers, PAA, dopamine, and iron ions. Upon UV irradiation, the hydrogel lost adhesion due to photoreduction of Fe^3+^ and oxidation of carboxy groups on cellulose and hydroxyl groups on dopamine to aldehydes, disrupting the gel network. When exposed to air, the material re-oxidize and regained its adhesive properties. Adhesion could be finely tuned by adjusting the intensity and duration of UV exposure. The resulting TENG retains these UV-reversible adhesion properties.

Visible-light-responsive electronic skins further expand the optical functionality of triboelectric systems. Liu et al. [4] developed a triboelectric electronic skin using poly(3-hexylthiophene) (P3HT) nanofibers embedded into PDMS composites as stretchable semiconductors, with AgNWs-doped PDMS as stretchable electrodes. This simple, single-sensor membrane leveraged the photoresponsive behavior of the semiconductor to convert visible light into electrical signals. The output varied with light intensity, enabling non-contact optical control and application in visible-light-guided robotics and smart systems.

In summary, stretchable conductive gel-based triboelectric sensors are continuously engineered to exhibit a wide range of advanced optical and multifunctional characteristics, many of which complement and extend the capabilities of human skin. These developments significantly broaden the potential of skin-like wearable TENGs for future applications.

### 4.4. Summary and Design Implications

Overall, stretchable conductive gel-based triboelectric sensors have been engineered to exhibit a diverse set of mechanical, self-healing, and optical/multifunctional properties, enabling their adaptation to skin-like, deformable, and dynamic operating environments. Advances in network design, dynamic bonding, and composite structuring have provided effective routes to achieving large stretchability, damage tolerance, and functional robustness. In parallel, the incorporation of optical and stimuli-responsive elements introduces additional modalities, such as visualized sensing, photothermal modulation, and light-responsive signal transduction. Together, these material- and structure-level developments extend the role of gel-based TENGs beyond mechanical energy harvesting toward multifunctional wearable platforms that integrate sensing, visualization, and human–machine interaction. These property-level considerations establish a foundation for the durability optimization and application-oriented designs discussed in the following sections.

## 5. Long-Term Stability of Gel-Based Triboelectric Sensors

Long-term operational stability is a prerequisite for the practical deployment of gel-based triboelectric sensors in wearable and flexible electronics. Beyond achieving high initial output performance, these devices must maintain reliable electrical and mechanical functionality under continuous deformation as well as prolonged exposure to diverse environmental conditions, including low and high temperatures, dryness, and humidity. As discussed in previous sections, conductive gels may exhibit limited temperature tolerance and are susceptible to solvent loss or uptake. In dry and hot environments, hydrogel-based TENGs may dehydrate, shrink, and lose mass, whereas under humid conditions, they can absorb moisture, swell, and gain weight. Such material-level variations can propagate into changes in device geometry and interfacial contact, ultimately influencing output stability. In parallel, repeated mechanical deformation and interfacial instability between gels and triboelectric layers constitute another important source of performance degradation. Accordingly, this section focuses on recent progress in enhancing the environmental adaptability and long-term durability of gel-based triboelectric sensors.

### 5.1. Gel-Based Triboelectric Sensors Operating in Extreme Environments

To ensure reliable operation in harsh environments, gel-based triboelectric sensors must tolerate wide temperature windows and resist performance degradation induced by freezing or heating. Considerable efforts have therefore been devoted to developing frost-resistant and heat-tolerant gel systems through solvent engineering, salt incorporation, and the design of ionogels or deep eutectic gels.

#### 5.1.1. Frost-Resistant Triboelectric Sensors

In early designs, water was commonly used as the solvent in conductive gels for fabricating TENGs. However, under sub-zero temperature conditions, such TENGs typically exhibit pronounced performance degradation. This behavior mainly originates from the limited water-retention capacity of hydrogels and the tendency of water to crystallize at low temperatures, which leads to the loss of elasticity and ionic conductivity [101].

To address this issue, researchers have explored the incorporation of ionic salts into hydrogels to enhance frost resistance. When dissolved, ionic salts modify the state of free water within the gel network and suppress ice formation. Meanwhile, they disrupt the hydrogen-bonding network among water molecules and strengthen interactions between water and polymer chains, thereby inhibiting ice crystal growth [102]. In addition, ionic salts increase osmotic pressure, which reduces the tendency of water molecules to migrate and freeze. It should be noted, however, that excessive salt concentrations may weaken polymer–polymer hydrogen bonding and consequently reduce mechanical strength.

Bao et al. [103] synthesized a hydrogel via one-step radical polymerization of acrylamide in a hydroxyethyl cellulose aqueous solution containing lithium chloride (LiCl). The resulting TENG remained unfrozen at −69 °C and exhibited nearly unchanged electrical output from room temperature down to −20 °C. With increasing LiCl concentration from 0 to 3 M, the hydrogel phase-transition temperature decreased from −11 °C to −62 °C, demonstrating tunable freezing behavior. Zhang et al. [104] developed a frost-resistant double-network conductive hydrogel based on glucan and Fe–COO^−^ coordinated poly(2-hydroxyethyl acrylate-co-acrylic acid) and constructed a corresponding TENG (GP-TENG). The introduction of LiCl generated hydrated lithium ions that bound free water molecules through hydrogen bonding, thereby enhancing antifreeze capability. The GP-TENG exhibited good durability, with its open-circuit voltage remaining virtually unchanged after 5000 contact–separation cycles. Moreover, GP-TENG maintained an open-circuit voltage of 170 V at −18 °C and 163 V after one month at that temperature, placing it among the high-performing frost-resistant TENGs reported to date.

Beyond salt-based strategies, organic solvents have been introduced into hydrogels through solvent exchange, in which water is partially replaced by organic molecules to lower the freezing point [105]. Organic solvents can form stronger and more diverse hydrogen bonds (e.g., via carboxyl, amino, and hydroxyl groups) with water, thereby weakening water–water interactions and suppressing ice crystallization [106]. Furthermore, van der Waals forces, hydrogen bonding, and dipole interactions among mixed solvents can cooperatively contribute to further freezing-point depression.

Zhu et al. [107] prepared a sodium alginate–acrylamide double-network hydrogel and replaced water with Gly to obtain a transparent and stretchable organic hydrogel (SPOH). The corresponding SPOH-based O-TENG operated stably at −20 °C for 14 days, with only a slight decrease in open-circuit voltage compared to room temperature. The Gly–water binary solvent system, together with the double-network structure, contributed to improved mechanical robustness and antifreeze performance.

**Figure 4:**
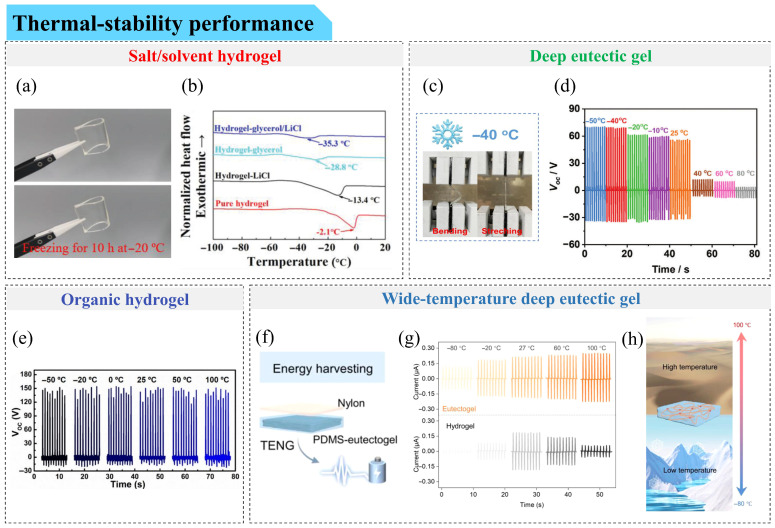
Thermal stability performance of gel-based TENGs. (**a**,**b**) Salt/solvent hydrogel. (**a**) Photos of Gly/LiCl-containing hydrogels before and after being frozen at −20 °C for 10 h. (**b**) DSC curves of hydrogels with different components [108]. Copyright © 2022 John Wiley & Sons, Inc. or related companies. (**c**,**d**) Deep eutectic gel. (**c**) Image showing bending and stretching of a deep eutectic gel at −40 °C. (**d**) Comparison of the open-circuit voltage of E-TENG at different operating temperatures [110]. Copyright © 2023 John Wiley & Sons, Inc. or related companies. (**e**) Organic hydrogel. (**e**) Open-circuit voltages of OHS-TENG at different temperatures [116]. Copyright © 2021 American Chemical Society. (**f**–**h**) Wide-temperature deep eutectic gel. (**f**) Schematic of a TENG based on deep eutectic gel. (**g**) Short-circuit current of TENG prepared based on hydrogel and deep eutectic gel at different temperatures. (**h**) Schematic showing that deep eutectic gels can be applied from −80 °C to 100 °C [117]. Copyright © 2024 American Chemical Society.

Combining ionic salts with solvent replacement can further enhance frost resistance. Guo et al. [108] developed a hydrogel by introducing LiCl and partially replacing water with Gly. As shown in Figure 4a, the AD-TENG retained transparency and flexibility after 10 h at −20 °C. The freezing point decreased to −13.4 °C with LiCl alone and further to −35.3 °C after Gly substitution, which was attributed to the colloidal effect of LiCl and strong hydrogen bonding between Gly and water (Figure 4b). Pushing this strategy further, Dai et al. [109] introduced both ethylene glycol and Gly into a PVA–PAM hydrogel doped with graphene oxide, yielding the GPPD-TENG. This device maintained stable open-circuit voltage and charge density over a temperature range from −60 °C to 20 °C, indicating favorable frost resistance.

In addition to organohydrogels, deep eutectic gels have emerged as another promising antifreeze platform. Lu et al. [110] prepared deep eutectic gels using ZnCl_2_, acrylic acid, ethylene glycol, and hydroxypropyl cellulose (HPC). As shown in Figure 4c, the gels remained bendable and stretchable at −40 °C. They exhibited ionic conductivities of 6.6 mS m^−1^ at −30 °C and 126.7 mS m^−1^ at 60 °C, and TENGs based on these gels achieved an open-circuit voltage of 69.7 V at −50 °C (Figure 4d).

Beyond partial solvent replacement, complete substitution of aqueous solvents with organic solvents has also been explored to improve low-temperature stability. Jing et al. [21] photo-crosslinked 4-acryloylmorpholine and acrylic carbonate (freezing point: −49 °C) with sodium perchlorate to form an organogel electrode. When combined with PDMS, the resulting Og-TENG retained flexibility and output voltage after five days at −20 °C, comparable to its performance at 25 °C. Nevertheless, some organic solvents are volatile or become highly viscous at low temperatures, which may compromise long-term stability. To mitigate this issue, Jing et al. [111] further developed organogel electrodes using non-volatile propylene carbonate and encapsulated them in silicone to reduce solvent loss.

Ionic liquids inherently possess low freezing points, high ionic conductivity, and low flammability, making ionogels particularly attractive for antifreeze TENG applications [101]. Zhu et al. [37] employed 1-ethyl-3-methylimidazole ethyl sulfate and acrylic acid to fabricate antifreeze ionogels that remained unfrozen and flexible at −80 °C. Combined with silicone rubber, the resulting SI-TENG showed stable output from −20 °C to 60 °C. Xia et al. [38] prepared ionogels using poly(vinylidene fluoride-co-hexafluoropropylene) (PVDF-co-HFP) and [EMIM][TFSI]. The corresponding M-TENG exhibited only a moderate decrease in open-circuit voltage from 110 V at 25 °C to approximately 80 V at −20 °C.

A direct comparison among different gel systems was reported by Li et al. [112], who fabricated hydrogel-, organogel-, and ionogel-based TENGs using zinc compounds and various solvents. At −30 °C, the hydrogel-based device (H-TENG) showed low output (36 V) due to water freezing, whereas the organogel-based O-TENG exhibited a higher output of 190 V owing to the presence of ethylene glycol. However, at 80 °C, both H-TENG and O-TENG suffered from solvent evaporation, resulting in reduced outputs of 28 V and 76 V, respectively. In contrast, the ionogel-based SI-TENG maintained output voltages of 187 V and 259 V after 8 h at −30 °C and 80 °C, respectively, highlighting its superior thermal stability.

#### 5.1.2. Heat-Tolerant Triboelectric Sensors

In practical applications, it is also important for TENGs to perform reliably under high-temperature conditions while maintaining stable electrical output. Studies have shown that elevated temperatures can lead to decay of surface charges on the friction layers of TENGs [113,114,115]. This degradation can be attributed to several factors: (1) thermal electron emission, where charges on the surface of the friction layer escape into the surrounding environment during operation, reducing surface charge density; (2) temperature-induced variations in the dielectric constant of friction-layer materials, which adversely affect electrical performance; and (3) thermal softening or deformation of constituent materials.

For traditional hydrogels, evaporation and water loss at high temperatures represent major challenges. Device encapsulation has been explored to reduce evaporation; however, hydrogel-based TENGs still tend to exhibit noticeable performance degradation under prolonged heating. A more effective approach is solvent replacement—that is, replacing water with less volatile solvents—to mitigate solvent loss at elevated temperatures. Currently, organogels and ionogels are among the most widely investigated heat-tolerant gel systems. However, organogels and organic hydrogels formed through solvent replacement often exhibit insufficient ionic conductivity.

To address this limitation, Wu et al. [116] prepared hydrogels from PVA and acrylamide in the presence of NaCl, followed by solvent replacement using Gly to produce organic hydrogels (abbreviated as ANV hydrogels). Due to the Hofmeister effect and electrostatic interactions, sodium ions and hydrogen-bonding networks within the hydrogel formed efficient charge-transport pathways, thereby compensating for conductivity losses associated with solvent replacement. When combined with silicone rubber to form an OHS-TENG, the device maintained an open-circuit voltage of 150 V at 100 °C—nearly identical to that observed at −50 °C and room temperature (Figure 4e). This high-temperature tolerance is attributed to the stability of sodium-associated interactions (stronger than hydrogen bonds) and the reversibility of hydrogen bonding. Despite this progress, developing organogel-based TENGs with simultaneously high conductivity and thermal stability remains an important research objective.

Ionogels, on the other hand, offer an alternative route to address these challenges, as ionic liquids typically possess high boiling points, high ionic conductivity, and low volatility, enabling ionogel-based TENGs to operate across a broad temperature window. Lei et al. [39] developed conductive ionic hydrogels using 1-vinyl-3-butylimidazole bromide, polyvinylpyrrolidone, acrylamide (AAm), betaine (BA), and cellulose nanofibers (CNFs), and fabricated a corresponding TENG (PPAVC-BA-TENG) using Ecoflex as the friction layer. Electrostatic interactions among the ionic liquid, cellulose nanofibers, and betaine imparted the ionic hydrogel with resistance to water loss at high temperatures. As a result, PPAVC-BA-TENG operated at 80 °C with only a slight decrease in electrical output compared to room temperature.

Beyond purely heat-tolerant behavior, conductive gels capable of functioning and self-healing at elevated temperatures have also been reported. Dang et al. [96] introduced hydroxypropyl cellulose (HPC) into molten α-lipoic acid and added 1-butyl-3-methylimidazole chloride as a conductive agent to prepare double-network ionogels via melt polymerization. These ionogels were combined with commercial VHB to fabricate a TENG (I-TENG). The ionogels self-healed at 100 °C within 1 h, restoring more than 90% of their electromechanical performance. Owing to the thermal stability of HPC and the compact polymer network, I-TENG maintained an open-circuit voltage of 77.6 V after 8 h at 80 °C, which is comparable to its room-temperature performance of 78 V.

Deep eutectic gels have also demonstrated promising heat tolerance. Yang et al. [117] synthesized a transparent deep eutectic solution (DES) using [2-(methacryloyloxy)ethyl]dimethyl-(3-sulfopropyl) (DMAPS) and acrylic acid (AA) as hydrogen-bond acceptor and donor, respectively, followed by photopolymerization to obtain deep eutectic gels. When integrated with PDMS, the resulting TENG (Figure 4f) maintained stable electrical output over a wide temperature range, including a steady current of ~0.2 μA at 100 °C (Figure 4g). The deep eutectic gels further exhibited thermal stability from −80 °C to 100 °C, which is associated with the non-volatility and inherent thermal robustness of the DES (Figure 4h).

In addition to sustained operation under elevated temperatures, resistance to direct thermal shock or combustion has also been examined. Li et al. [118] prepared conductive organic hydrogels from N-hydroxyethyl acrylamide, carrageenan, lithium chloride, and Gly, and combined them with Ecoflex to fabricate PCLG-TENG. In this system, the hydrogel acted as a thermal buffer, absorbing heat and protecting the Ecoflex layer. When exposed to an alcohol flame for 30 s, the open-circuit voltage of PCLG-TENG remained nearly unchanged and decreased by only ~20% after 120 s of continuous burning.

### 5.2. Durability of Gel-Based Triboelectric Sensors

While self-healing focuses on damage recovery, durability emphasizes long-term retention of electrical and mechanical performance under continuous deformation and environmental exposure. When used over extended periods, TENGs often encounter challenging environmental conditions such as dryness, high humidity, cold, and heat. As previously discussed, conductive gels exhibit limited temperature tolerance and are susceptible to solvent loss. In dry and hot environments, hydrogel-based TENGs may gradually dehydrate, shrink, and lose mass, whereas under humid conditions, they can absorb moisture, swell, and gain weight. Such volume and mass variations at the material level can propagate into changes in device structure and interfacial contact, ultimately affecting the electrical output stability of TENGs. In addition, repeated mechanical deformation and interfacial instability between gels and triboelectric layers represent another major source of performance degradation.

#### 5.2.1. Cyclic Stability and Interface Integrity of Gel-Based Triboelectric Sensors

Interfacial delamination represents a major challenge for the practical application of gel-based triboelectric sensors. This issue primarily originates from mismatched surface chemistry and dynamic mechanical behavior between hydrophilic gel layers and commonly used hydrophobic elastomeric triboelectric materials such as PDMS and Ecoflex. As a result, interfacial adhesion is often dominated by weak physical interactions, which are prone to failure under repeated or prolonged deformation, leading to signal instability or device degradation [119,120].

Previous studies have shown that both gel curing behavior and interfacial microstructure strongly influence interfacial stability. Excessively smooth elastomer surfaces restrict wetting and anchoring of pre-gel solutions, whereas introducing micro-/nanoscale roughness or hierarchical structures increases effective contact area and promotes mechanical interlocking during in situ gel solidification [121,122]. For example, Pandey et al. [123] encapsulated a PEO/LiTFSI ionic gel within Ecoflex through in situ curing, forming a hierarchical interfacial architecture that effectively mitigated delamination and enabled stable operation under tensile strains up to 375%.

Beyond morphological regulation, integrated construction strategies aim to eliminate laminated interfaces by forming continuous conductive and mechanical networks. Han et al. [124] spray-coated Ag nanowires onto an ionic gel surface, generating hydrogen-bond-assisted interfacial coupling and a monolithic device architecture, which exhibited low interfacial resistance and stable output under 80% tensile strain.

Nevertheless, under long-term cyclic deformation, polymer chain relaxation and interfacial molecular mobility may still induce gradual adhesion decay, particularly in systems relying solely on physical interactions. Therefore, further reinforcement through interfacial chemical coupling and synergistic material design is often required. Overall, these studies indicate that enhancing gel–elastomer interfacial stability relies on the synergistic optimization of interfacial microstructure, curing behavior, and device construction, while maintaining compatibility with triboelectric charge generation.

#### 5.2.2. Anti-Drying Gel-Based Triboelectric Sensors

Two main strategies have been developed to enhance the drying resistance of stretchable conductive gel-based TENGs: (1) improving the solvent retention capacity of the gel itself and (2) isolating the gel from the environment through encapsulation.

**Figure 5:**
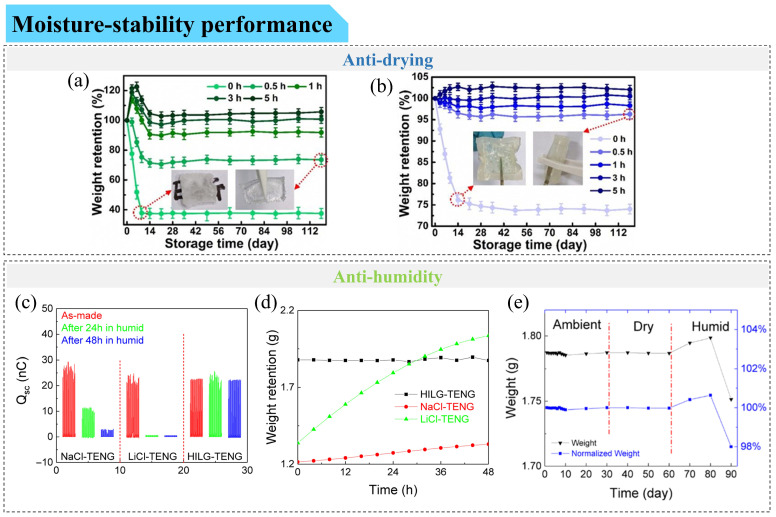
Moisture-stability performance of gel-based TENGs. (**a**,**b**) Anti-drying performance. (**a**) Water content of AVN gels after soaking in Gly for different times. (**b**) Water content of AVN-TENG after 120 days of storage [116]. Copyright © 2021 American Chemical Society. (**c**–**e**) Anti-humidity performance. (**c**) Comparison of Qsc and (**d**) normalized weight holding rate of TENGs prepared from PAAm hydrogels containing HILG, LiCl, and NaCl after 48 h in a humid environment. (**e**) Weight holding rate and normalized weight holding rate of HILG-TENG at 20 °C under different humidities (20%, 40%, and 80%) [101]. Copyright © 2020 American Chemical Society.

Hou et al. [125] reported KMGHCa gels using gellan gum and calcium chloride, poly(N-hydroxyethyl acrylate), and 3-(trimethoxysilyl) propyl methacrylate (KH570)-modified MXene (K-MXene) as fillers. After 3 days at 25 °C and 45% relative humidity (RH), the water loss of the KMGHCa gel was 16.5%, which is attributed to the incorporation of calcium chloride salt. When encapsulated with Ecoflex to form KMGHCa-TENG, water loss decreased to 1.4%. Wang et al. [126] utilized two hygroscopic salts—calcium chloride and zinc chloride—to synthesize anti-drying, double-network cellulose/PVA/borax (CPB) hydrogels. At 20–24 °C and 40–50% RH, the hydrogel exhibited near-zero water loss for 30 days. After being encapsulated in Ecoflex, CPB-TENG maintained 96% of its initial output after 200 bending cycles in a fume hood. One drawback is that under high-humidity conditions, these same salts can cause water uptake, leading to swelling of the TENG. In addition to salt incorporation, solvent displacement offers another effective strategy. Wu et al. [116] partially replaced water in PAM/PVA/NaCl hydrogels (AVN) with Gly, effectively improving drying resistance. AVN gels were soaked in Gly for 0–5 h, and the longer the soaking time, the better the anti-drying of the AVN gels (Figure 5a). At 25 °C and 30% RH, TENG based on AVN hydrogels retained over 95% of their weight after 120 days, compared to 75% after just 14 days for untreated samples (Figure 5b). However, the tradeoff of this approach for the extended lifetime is reduced electrical output due to the replacement of water with organic solvents.

#### 5.2.3. Anti-Humidity Gel-Based Triboelectric Sensors

Studies have shown that rising humidity decreases TENG output [127], mainly due to increased absorption of water molecules on the friction layer, which leads to triboelectric charge loss [128]. For skin-mounted flexible triboelectric sensors, sweating is a common and persistent challenge, making moisture resistance critical.

A widely adopted strategy is to encapsulate the stretchable conductive gel to shield it from environmental humidity and sweat. Lv et al. [101] developed ionogels using 1-butyl-2,3-dimethylimidazolium bis(trifluoromethylsulfonyl)imide and further prepared TENG. After 48 h of storage in a humid environment, the Qsc of HILG-TENG remains largely unchanged (Figure 5c). Compared with the NaCl- and LiCl-prepared TENG, the weight of HILG-TENG did not change much after 48 h of storage in a humid environment (Figure 5d). In addition, in order to investigate the long-term stability of HILG-TENG, the weight holding rate and normalized weight holding rate of HILG-TENG were obtained after 90 days of continuous testing in various humidity environments (Figure 5e), indicating strong resistance to both drying and swelling. Firdous et al. [86] reported SHE-TENG using self-healing PAA-GaF hydrogels (made from acrylic acid, gum Arabic and Fe^3+^) combined and encapsulated with Ecoflex. Notably, the hydrogel self-heals in dry, wet, and frozen states, with 91% self-healing efficiency retained underwater. SHE-TENG retained its output power of 7.28 W/m^2^ after 24 h in ionized water and showed minimal performance degradation after 18 days at 22 °C and 10% RH. Additionally, improving the surface hydrophobicity of friction layers also enhances moisture resistance [129]. Methods include engineering 3D micro- or nanostructures, introducing hydrophobic functional groups (e.g., ester groups), or incorporating hydrophobic nanoparticles into the surface of the layer [130]. Qian et al. [131] drew inspiration from octopus tentacles to design a honeycomb micro-pattern on an Ecoflex surface and further prepared it into a TENG. It promoted water drainage and enhanced the electrical output of a gel electrode-based TENG in a humid environment. Park et al. [132] developed DOA5-TENG using plasticized PVC gel with dioctyl adipate (DOA), a hydrophobic plasticizer. DOA5-TENG showed minimal weight changes in water for 10 days and a high temperature of 65 °C for 30 days and maintained an open circuit voltage of 198 V for 30 days at 65 °C, suggesting its moisture resistance and heat tolerance.

### 5.3. Summary of Long-Term Stability

This section summarizes recent progress in enhancing the long-term stability of gel-based triboelectric sensors under diverse environmental and mechanical stresses, including low and high temperatures, cyclic deformation, drying, and humid conditions. Current strategies mainly rely on solvent engineering (e.g., ionic liquids, organic solvents, and deep eutectic systems), interfacial microstructure regulation, and device encapsulation to mitigate performance degradation. Despite these advances, most reported approaches address specific stability issues rather than providing comprehensive robustness against multiple simultaneous stressors. In addition, trade-offs between ionic conductivity, mechanical compliance, and environmental tolerance are frequently observed. The lack of standardized testing protocols further complicates direct comparison among different systems. These observations indicate that achieving broadly stable gel-based TENGs remains a nontrivial challenge.

## 6. Configurations of Triboelectric Devices Based on Conductive Gels

### 6.1. Thin-Film Triboelectric Sensors

**Figure 6:**
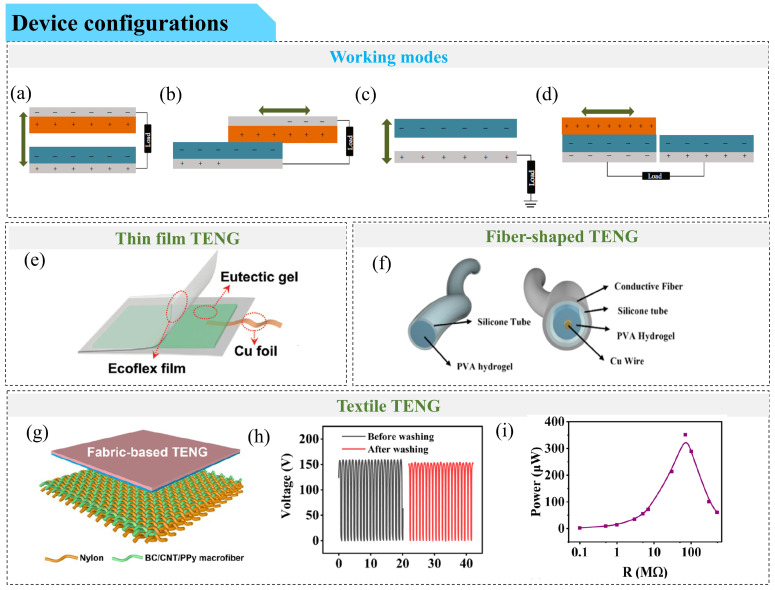
Device configurations of gel-based TENGs. (**a**–**d**) Operation modes of triboelectric nanogenerators: (**a**) vertical contact–separation mode, (**b**) contact-sliding mode, (**c**) single-electrode mode, and (**d**) freestanding triboelectric-layer mode. (**e**) Thin-film configuration: schematic diagram of a thin-film TENG [110]. Copyright © 2023 John Wiley & Sons, Inc. or related. (**f**) Fiber configuration: morphology of tube hydrogel TENG (3 cm) with pure PVA as electrode (single-electrode mode) and positive tribolayer (contact-separation mode) [140]. Copyright © 2024 American Chemical Society. (**g**–**i**) Textile configuration: (**g**) schematic diagram of fabric-based TENG structure, (**h**) output voltage of the fabric-based TENG before and after washing, and (**i**) instantaneous power versus external load resistance [157]. Copyright © 2022 Springer Nature.

The majority of the gel-based TENGs developed by now have a thin-film configuration. It usually has four working modes: vertical contact-separation mode (Figure 6a), contact-sliding mode (Figure 6b), single-electrode mode (Figure 6c) and freestanding triboelectric-layer mode (Figure 6d). Significant progress has been made to improve their output performance and increase their multifunctionality through materials engineering [26,86]. As a result, many thin-film TENGs demonstrated high performance in electrical outputs, flexibility, and even self-healing capabilities [133,134,135,136,137]. Through thickness control and the introduction of micro- and nanostructures, the sensitivity and flexibility of the thin-film TENGs were greatly improved [110]. The schematic diagram of the TENG is shown in Figure 6e.

However, thin-film TENGs still face several limitations. For instance, their low breathability can hinder skin respiration and cause discomfort when worn on human skin for a prolonged duration. Additionally, the typical thickness [138] of current configurations—often at the millimeter scale—limits their ability to conform to complex three-dimensional surfaces, making them unsuitable for large-area coverage on dynamic or irregular structures, such as the full body of a humanoid robot. To improve intrinsic elasticity, the thickness of the thin-film TENGs needs to scale down to the micron scale into ultra-thin TENGs. However, such ultra-thin films are prone to manufacturing defects, inconsistency, and mechanical damage during handling and electrical breakdown during operation, presenting significant challenges for practical applications [139]. On the contrary, fiber-based [140] and textile-based [141] TENGs offer unique advantages, including superior breathability, mechanical compliance, intrinsic elasticity and resistance to the washing cycles, providing better wearing comfort and making them more suitable for full-body, wearable electronics and conformal systems. The schematic diagram of the TENG is shown in Figure 6f.

### 6.2. Gel-Based Fiber-Shaped Triboelectric Sensors

To address the structural and functional limitations of thin-film TENGs, fiber-shaped triboelectric nanogenerators (F-TENG) have emerged as promising alternatives [142]. The core strategy in fabricating F-TENGs lies in integrating the electrode and friction layers within a one-dimensional fibrous structure. However, precursor solutions of hydrogels and their mixture often suffer from poor spinnability and limited strength, posing challenges in fiber formation [143]. Several methods for preparing gel fibers have been demonstrated in the lab, including batch-based techniques and some continuous approaches. Batch-based approaches include capillary polymerization [144] and draw spinning [145,146], while continuous methods comprise continuous wet spinning [147,148], melt spinning [149], microfluidic spinning [150], fiber extrusion (e.g., during that of 3D printing) [151], and electrospinning [152]. Among them, continuous wet spinning and melt spinning are the most commonly adopted for gel fiber spinning due to their reliability and compatibility with large-scale industrial manufacturing processes.

TENGs operate in four fundamental modes: single-electrode, freestanding triboelectric layer, vertical contact-separation, and lateral sliding. Gel-based single-fiber F-TENGs typically operate in either the single-electrode or vertical contact-separation mode [153]. A typical single-fiber F-TENG features a coaxial structure, comprising a conductive gel-based core electrode and a triboelectric sheath layer.

Researchers have adopted injection and coating as two commonly used methods for preparing gel fiber-based F-TENG. The injection method starts with a hollow sheath tube of the friction layer, while the coating method starts with the gel electrode core fiber. Jing et al. [111] mixed 4-acryloylmorpholine (ACMO) monomer with propylene carbonate solvent to prepare a precursor solution for organic embryonic electrodes and injected it into a transparent silicone hollow fiber mold, followed by optical crosslinking to fabricate a core–shell structured F-TENG (GS fiber). The resulting GS fiber exhibited remarkable flexibility and a tensile strain exceeding 700%, effectively mitigating the cracking issue seen in metal-based electrodes and the leaking issue seen in liquid-based electrodes. Shuai et al. [143] coated poly(methyl acrylate) (PMA) onto PNA hydrogel fibers (copolymers of acrylamide and N-acryloyl glycinamide), fabricating a PNA/PMA core-sheath F-TENG with a high stretchability of up to 900% as well as a measurable electrical conductivity of 0.69 S/m. Notably, these fibers could recover 85% of their mechanical strength within 10 min at 60 °C. Xiao et al. [140] encapsulated PVA hydrogels in silicone tubes and doped them with graphite carbon nitride (g-C_3_N_4_) to enhance conductivity. Their spiral disc-shaped TENG produced 25 V open-circuit voltage and 1 μA short-circuit current, significantly outperforming the 1 V output voltage from its tubular counterpart due to increased contact surface area. Integrating single-fiber F-TENG into textile is another way to effectively scale up contact areas and the electrical output of wearable F-TENG devices [154].

### 6.3. Gel-Based Triboelectric Textiles

Conventional textile-based TENGs have been explored rapidly, offering advantages such as breathability and mechanical compliance over planar thin-film configurations. However, most existing designs still incorporate rigid or semi-rigid metal-based conductive components (e.g., braided stainless steel, copper fibers, metal-coated fibers and metal-embedded elastomers) as electrodes [154]. Though highly conductive, these metal electrodes are heavy and expensive and often suffer from mechanical mismatch with soft textile substrates and skin, resulting in poor wearing comfort, interface damage, and reduced durability [155]. Moreover, metal fibers are prone to fatigue, corrosion and cracking during deformation or laundry cycles, limiting their long-term reliability in wearable scenarios. In contrast, gel-based electrodes utilize soft, intrinsically stretchable conductive gel electrodes—such as hydrogels, organogels, and ionogels—to replace rigid metallic electrodes. These gels provide significant advantages in terms of lightweight, low cost, intrinsic flexibility and compatibility with skin-like or deformable surfaces [156].

Recent studies have demonstrated various gel-based textile TENGs (T-TENGs) and textile architectures with promising triboelectric performance. For instance, Hu et al. [157] developed strong, biodegradable hydrogel fibers integrating bacterial cellulose (BC), carbon nanotubes (CNTs), and polypyrrole (PPy) by wet-stretch and wet-twist. The schematic diagram of the fabric-based TENG structure is shown in Figure 6g. Fabric-based TENG achieving stable electrical output under 85% relative humidity and after multiple wash cycles (Figure 6h). In addition, the fabric-based TENG can have a maximum output power of 352 μW and can power electronic devices (Figure 6i). Dong et al. [141] fabricated a textile-based TENG containing an organic hydrogel electrode inside a silicone tube. The organic hydrogel electrodes were made by injecting precursor solutions containing poly(vinyl alcohol), gelatin, glycerin, PEDOT:PSS, and sodium chloride into hollow silicone tubes. The resulting T-TENG achieved a strain of about 700% and robust performance down to −20 °C and resistance to contamination by liquids such as milk, juice, coffee and water. Moreover, textile-based TENG and wool fabrics are separated in contact, and the short-circuit current obtained is about 0.8 μA, and the electrical output performance is stable. Ionogels have further advanced the field by offering improved conductivity and stability. Based on ethyl acrylate (EA) and ionic liquid 1-ethyl-3-methylimidazolium bis(trifluoromethyl sulfonyl) imide ([EMIM][TFSI]), Zhong et al. [158] reported a stretchable ionogel fiber-based TENG (abbreviated as W-TENG). W-TENG can achieve a strain of 400% and maintain stable electrical output performance at temperatures ranging from −18 °C to 200 °C. More recently, Tan et al. [149] developed covalently crosslinked ionogel fibers (DOU-IG). DOU-IG has intrinsic self-healing capability, high environmental stability and a maximum power output of 80.8 mW/m^2^. The ionogel fiber was based on 1-ethyl-3-methylimidoline bis(trifluoromethylsulfonyl)imide and polyurethane with dynamic dimethylglyoximeurethane (DOU) covalent crosslinks. In addition, as previously described, Shuai et al. [143] reported PNA/PMA core-sheath F-TENG. It operates primarily through a contact-separation single-electrode mode. In addition, the maximum peak power density reaches 88 mW/m^2^, fully demonstrating the application potential of fabric-based TENG.

Despite these advances, most gel-based textile TENGs are still in the early stages of development, particularly regarding their available gel fiber types, large-scale integration, manufacturability, lifetime, and end-of-life sustainability. Nonetheless, they represent a critical step toward replacing bulky, rigid metal-based electrodes in textile-based TENGs.

### 6.4. Summary of Device Configurations for TENGs

Overall, conductive gel-enabled triboelectric devices have evolved from conventional planar thin-film architectures toward one-dimensional fiber-shaped generators and further into textile-integrated systems, reflecting a clear structural progression toward higher conformability, breathability, and system-level wearability. Thin-film TENGs remain advantageous for fundamental studies and multifunctional integration, while gel-based fibers and textiles offer improved mechanical compliance, scalable contact area, and better compatibility with large-area and full-body wearable platforms.

At the same time, the transition from films to fibers and textiles introduces new challenges associated with gel spinnability, structural uniformity, interface robustness, and device integration consistency. Current reports demonstrate the feasibility of these configurations, yet most examples remain at the proof-of-concept stage. Further systematic efforts in materials design, fiber-processing strategies, and textile-level integration are required to establish reliable, reproducible, and application-oriented gel-based triboelectric device architectures.

## 7. Applications of Gel-Based Triboelectric Sensors

Triboelectric energy generators show great potential for harvesting mechanical energy from sources like body movement, water droplets, and ocean waves. Researchers have developed various integrated systems using these devices, such as self-charging units, light-emitting displays, and heat-activated wound-healing patches. In many reported cases, gel-based TENGs exhibit relatively lower electrical output than devices employing rigid metallic or carbon-based electrodes, and their charging rates toward energy storage units are often slower. Consequently, they are less suitable for high-power energy applications but are highly promising for low-power, flexible, and wearable applications—such as self-powered sensors, electronic skin, wound care devices, and human–machine interfaces, where comfort, flexibility, and multifunctionality matter more than energy output. In addition, single-electrode configurations are widely adopted owing to their simplified wiring and suitability for skin-mounted operation.

### 7.1. Biomedical Motion Detection

Stretchable conductive gel-based TENGs have demonstrated significant potential for real-time, wearable biomedical motion detection due to their mechanical compliance, high sensitivity to mechanical stimuli, and intrinsic stretchability. These characteristics make them suitable for non-invasive, continuous monitoring of physiological signals and human motions, which are critical for early disease detection, rehabilitation assessment, and human–machine interfaces.

**Figure 7:**
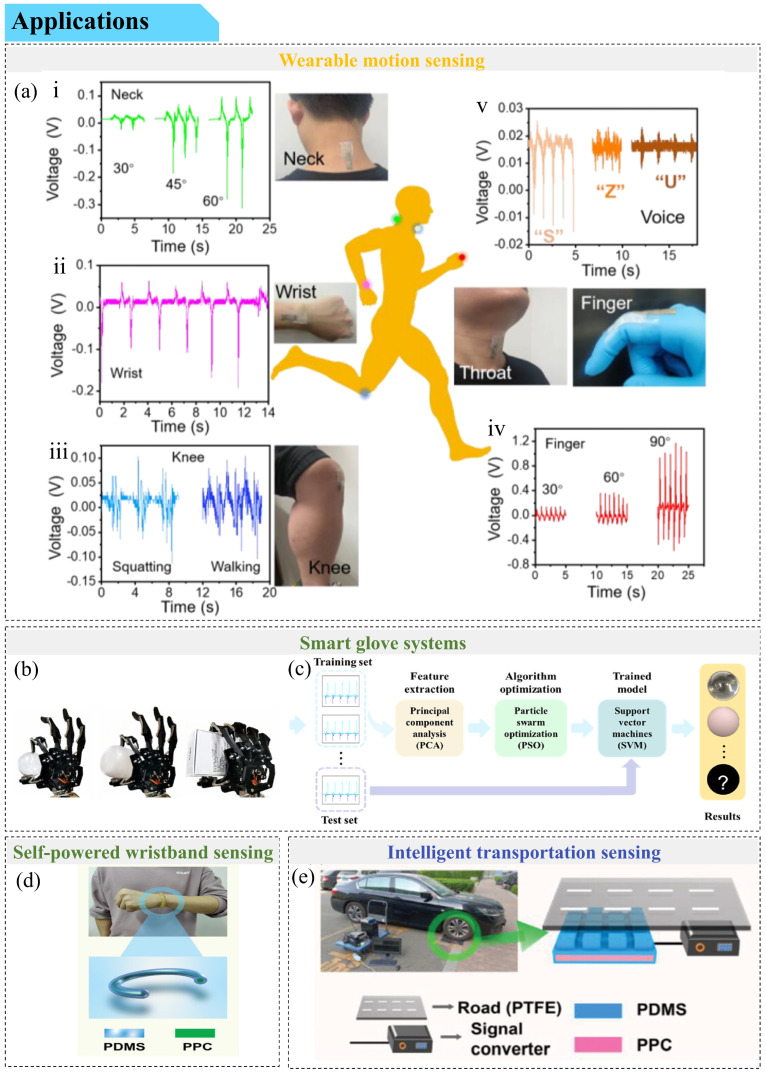
Representative applications of skin-like TENGs. (**a**) Wearable motion sensing: application demonstration of stretchable skin-like TENG of finger flexion (**i**), wrist rotation (**ii**), neck tilting (**iii**), knee dynamics (**iv**), swallowing and vocal cord movements (**v**) [133]. Copyright © 2022 MDPI. (**b**,**c**) Smart glove systems: (**b**) controlling a robotic hand to grab spheres, cylinders, and cuboids, and (**c**) PTSM-TENG fixed at the front end of the glove for object identification [161]. Copyright © 2025 American Chemical Society. (**d**,**e**) Intelligent transportation sensing: (**d**) PPC-TENG self-powered sports wristband, and (**e**) schematic diagram of PPC-TENG for vehicle-signal detection [171]. Copyright © 2023 John Wiley & Sons, Inc. or related companies.

To demonstrate these capabilities, Figure 7a illustrates a representative skin-like TENG platform achieving seamless conformal contact with diverse body surfaces. Regardless of the underlying active materials, such architectures excel at capturing a broad spectrum of signals, ranging from vigorous joint articulations (e.g., finger flexion, wrist rotation, neck tilting, and knee dynamics) to subtle physiological vibrations (e.g., swallowing, and vocal cord movements) [133]. This extensive sensing repertoire underscores the potential of skin-like interfaces—a domain where conductive gel-based TENGs offer distinctive advantages due to their inherent soft-tissue-like modulus and superior biological integration.

Rahman et al. [159] developed a hydrogel-based TENG (ZPcHLH-TENG) from LiCl, acrylamide, hydroxyethyl acrylates, and zeolite imidazolate framework-8 (ZIF-8) nanofillers, encapsulated with surface-textured Ecoflex. This device successfully monitored movements of the fingers, wrists, hands, and feet and distinguished between various gait patterns, including slow walking, fast walking, jogging, and jumping. When integrated with a microcontroller, it enabled interactive applications such as virtual gaming interfaces, demonstrating potential utility in rehabilitation, motion tracking, and human–machine interfaces. Zhu et al. [37] fabricated ionogel-based TENG (SI-TENG) from ionic liquid [EMIm][EtSO_4_], PAA, nano-Fe_3_O_4_ and amino-terminated hyperbranched polyamide (NH_2_-HBP), encapsulated with SiO_2_-modified silicone rubber. This self-powered sensor could monitor not only joint flexion but also subtle movements in the throat region, such as swallowing, nodding, and head shaking, relevant for speech therapy and swallowing disorder monitoring. Wu et al. [160] prepared a deep eutectic gel-based TENG (E-TENG) from sodium lignosulfonate (SL), ferric chloride hexahydrate (FeCl_3_·6H_2_O), acrylic acid (AA), and ammonium persulphate (APS), encapsulated with Ecoflex. The device exhibited good sensitivity under various environmental conditions, including sub-zero temperatures (−20 °C) and underwater, while enabling wireless transmission of motion signals to mobile devices. This highlights its potential in harsh-environment physiological monitoring. Zhang et al. [161] developed an ionic hydrogel-based TENG (PTSM-TENG) from polypropylene amine, tannic acid (TA), sodium alginate and MXene, encapsulated with silicone rubber, and integrated it into a smart glove with a microcontroller. As shown in Figure 7b, the system could perform quantitative detection of finger bending angles and enable gesture recognition and remote object manipulation via Bluetooth, demonstrating potential in neuromuscular rehabilitation and prosthetic control. In addition, the system can also be combined with machine learning to recognize objects (Figure 7c). Lu et al. [110] fabricated a TENG using hydroxypropyl cellulose-based deep eutectic gels (HPC-MDES) combined with Ecoflex. The device detected not only large movements but also subtle facial expressions (e.g., frowning, smiling, and blinking) and respiratory rhythms. A glove with five TENG sensors and a microcontroller enabled real-time tracking of finger gestures and actuator control. Qu et al. [162] designed an ionic hydrogel-based wearable strain triboelectric sensor skin (STSS) from poly(vinyl alcohol), xanthan gum, NaCl, and glycerin, topped with silicone rubber. When incorporated into a two-finger soft robotic gripper, the sensors could detect object size and object motion, such as slippage, and function reliably underwater.

Integration of TENG sensors with machine learning enhances signal interpretation accuracy. Li et al. [163] fabricated a PHM-TENG using MXene-based hydrogels made from acrylamide, hydroxypropyl methylcellulose and MXene (Ti_3_C_2_Tx), combined with VHB tape. The device was capable of recognizing handwritten letters with 96.7% accuracy with the help of deep learning, indicating promise for neuromuscular diagnostics and motor assessment. Han et al. [164] prepared anti-freezing conductive gels by polymerizing acrylamide (PAM) and the zwitterionic [2-(Methacryloyloxy)ethyl]dimethyl-(3-sulfopropyl)ammonium hydroxide (SBMA) in a glycerol/water mixed solvent with added NaCl and gelatin, and further created TENGs with Ecoflex. By integrating them into insoles, they can be used to monitor skiing activities. When combined with the random forest algorithm from machine learning, the recognition accuracy for 11 different skiing movements reached 97.1%, demonstrating great potential in sports monitoring. Wang et al. [165] fabricated a graphene oxide-polyacrylamide hydrogel as the friction layer for a TENG integrated into smart insoles, achieving up to 99.5% accuracy in gait recognition with the support of machine learning. The system also identified abnormal gait patterns in Parkinson’s and hemiplegia patients, aiding in rehabilitation and health monitoring. Luo et al. [166] developed a NaCl-PVA hydrogel-based TENG and integrated four of them into a neckband device. The device distinguished between talking, coughing, turning the head, and nodding with over 96% accuracy, supported by machine learning. When paired with seatbelt sensors that detect the driver’s breathing and yawning status, the system enables multi-dimensional drive fatigue monitoring.

### 7.2. Tactile Perception

Tactile perception covers a wide range of haptic sensing abilities, including static and dynamic force sensing, slip detection, texture discrimination, and object recognition based on haptic data. For static and dynamic force sensing, Zhu et al. [167] developed DES-TENG by combining stretchable conductive hydrogels—synthesized from maleic acid, acrylamide, and polyethylenimine—with silicone rubber. When integrated with a microcontroller and placed at the sole of a shoe, the DES-TENG enabled a human gait sensing system capable of detecting both step count and contact pressure, demonstrating sensitivity suitable for high-impact sports such as triple jump or hurdling. For slip detection haptics, Liu et al. [168] designed a highly stretchable triboelectric touchpad by integrating an AHSL-TENG (fabricated by combining a conductive hydrogel from acrylamide, 2-hydroxyethyl methacrylate, and lithium chloride with sandpaper-textured Ecoflex) and liquid metal. This touchpad, connected to a Bluetooth signal transmission system and trained with a Transformer-based deep learning algorithm, achieved a 96.83% gesture recognition accuracy and enabled directional control of unmanned aerial vehicles (UAVs) through figure sliding, showcasing promising potential in sliding-based haptic applications. In object and texture recognition, Zhou et al. [169] synthesized CMPG-TENG by combining organic hydrogels—based on poly(vinyl alcohol), cellulose nanofibers, and MXene—with Ecoflex. The CMPG-TENG could distinguish handwritten letters at −35 °C and enabled encrypted communication. When connected to a robotic hand and wireless transmission module, it could detect the size and texture of objects at low temperature.

Stretchable conductive gel-based TENGs have also shown significant promise in various other applications. Bu et al. [170] developed OP-TENG containing ionogels based on PVA, bacterial cellulose (BC), and 1-allyl-3-methylimidazole chloride ([AMIM]Cl). OP-TENG exhibited good force sensitivity up to 50 N. By coupling OP-TENGs into a single-electrode mode SSEM-TENG and attaching it to the skin, it could recognize stroke sequences and stroke counts in written characters, suggesting its potential in sliding haptic sensors and human–computer interactions when combined with machine learning. In vehicle detection, Li et al. [171] prepared PPC-TENG by combining conductive hydrogels (based on PVA, PAAm and tannin-modified cellulose nanocrystals) with PDMS containing protrusion arrays on the surface. PPC-TENG can be made into a self-powered smart sports wristband for monitoring the driver’s physical activity (Figure 7d). Placed underground, PPC-TENG could sense pressure signals to identify traffic conditions and vehicle maneuvers without surveillance cameras. It provided real-time accident alerts via mobile phone notification systems (Figure 7e). Furthermore, due to partial substitution of water with Gly, the gel-containing device could operate stably from −30 °C to 40 °C. Gu et al. [172] developed IF-TENG by integrating ionogels (based on zinc oxide nanoparticles, 1-ethyl-3-methylimidazole ethyl sulfate and PAA) with fluoroplastic release film and nitrile rubber. The resulting pressure sensor was used to fabricate an attitude sensor capable of detecting vehicle or ship tilt angles. For instance, when tilted more than 20°, it triggered an alert via LED indicators, demonstrating its utility for transportation safety. In sports applications, Tian et al. [87] fabricated PAGCA-TENG comprising frost-resistant Gly-containing hydrogels (based on gelatin, silver nanowires, tannic acid-modified carbon nanotubes, and polyacrylamide). This device detected the status of curling stones (e.g., being hit or displaced) and can be used to detect foul play during the game. Additionally, Dai et al. [173] reported TENG for Braille recognition systems by integrating an organic hydrogel—composed of polyacrylamide, clay, potassium iodide and Gly—between two layers of polyazomethine membranes. When this sandwiched structure was brought into contact with Braille characters patterned on a PDMS film, the Braille characters activated the TENG sensor, and the system—coupled with amplifiers and microcontrollers—converted tactile inputs into sound output, providing an accessible interface for visually impaired individuals.

### 7.3. Other Emerging Applications

Skin-like, conductive gel-based TENGs have also found application in many emerging areas such as self-powered noncontact interactions [114,174,175], electro-tactile systems for AR/VR, and stimulated wound healing.

In a pioneering application, Shi et al. [88] developed a self-powered electro-tactile system using a TENG with spherical electrode arrays. The system controls induced currents such that discharge electrodes operate in a non-contact mode, allowing for painless electro-tactile feedback at a controlled distance (0.3–0.5 mm). Compared to needle or microneedle electrodes, the round electrodes offer gentler stimulation, making them more suitable for skin contact. Jeong et al. [176] developed an organic gel-based TENG and constructed a TENG textile (iTENG). This textile was demonstrated to stimulate wound healing by converting ambient energy into electrical stimuli and promoting fibroblast proliferation and differentiation. In vivo tests showed enhanced re-epithelialization, collagen formation, and neovascularization in mice, leading to accelerated wound closure with minimal scarring. 

Table 3 summarizes the representative performance metrics of conductive gel-based TENGs, highlighting a predominant preference for the single-electrode configuration in most reported cases, owing to its minimized wiring complexity for skin-integrated interfaces. While these devices demonstrate impressive versatility across applications ranging from human–machine interaction to underwater sensing, a critical analysis of the reported data suggests that operational durability appears to be correlated with solvent retention strategies. Although many systems showcase extended temperature windows and resistance to dehydration (e.g., through the use of non-volatile ionogels or hygroscopic additives), long-term stability under continuous dynamic deformation—often on the order of 10,000 cycles or higher—remains an important benchmark for practical wearable deployment. These comparisons suggest that intrinsic gel chemistry is an important factor in enabling reliable, all-weather gel-based electronic skins.

### 7.4. Summary of Applications

Overall, recent studies demonstrate that stretchable conductive gel-based TENGs have enabled a diverse range of application scenarios, spanning biomedical motion monitoring, tactile perception, human–machine interfaces, and emerging interactive and therapeutic systems. Across these demonstrations, the combination of soft gel electrodes, elastomeric triboelectric layers, and simplified single-electrode configurations provides a common materials and device foundation that supports conformal skin contact, mechanical compliance, and stable signal generation under deformation. While their electrical output is generally modest compared with rigid-electrode counterparts, gel-based TENGs consistently exhibit sufficient sensitivity, robustness, and functional adaptability for low-power wearable sensing and interaction tasks. Collectively, these application examples highlight the practical relevance of conductive-gel-enabled triboelectric devices as versatile building blocks for skin-like electronic systems.

## 8. Conclusions and Future Perspectives

This review provides a comprehensive and structured overview of stretchable conductive gel-based TENGs toward skin-like sensing applications, covering material design, key performance attributes, device configurations, and representative application scenarios. Different classes of conductive gels, including hydrogels, organogels, ionogels, and emerging gel systems such as deep eutectic gels, are systematically summarized and compared in terms of their composition, crosslinking strategies, conductivity, and mechanical characteristics. The pivotal role of conductive gels as stretchable electrodes in bridging skin-like mechanical compliance and triboelectric signal transduction is highlighted. Furthermore, the key performance attributes of gel-based skin-like triboelectric sensors—including stretchability, self-healing capability, environmental tolerance, electrical durability, and optical functionalities—are critically discussed together with typical thin-film, fiber-shaped, and textile-based device architectures. Collectively, these advances demonstrate that stretchable conductive gels constitute a promising material basis for constructing next-generation skin-like triboelectric sensing systems capable of continuous mechanical signal transduction.

Despite the significant progress achieved, several critical challenges must be addressed before stretchable, conductive, gel-based, skin-like TENGs can be translated into widespread practical use.

(1) Expanding gel material diversity and multifunctionality.

The current material landscape is still dominated by polyacrylamide-based hydrogels and a limited number of ionogels, which constrains the simultaneous optimization of conductivity, mechanical robustness, and environmental stability. Future research should focus on expanding gel chemistries by incorporating dynamic covalent networks, supramolecular interactions, and nanohybrid fillers to achieve broader tunability and multifunctionality. In addition, integrating natural polymers (e.g., chitosan, alginate, and cellulose derivatives) and bio-derived building blocks may further enhance biocompatibility and sustainability, providing new opportunities for bio-integrated and skin-conformal TENG systems.

(2) Enhancing device durability, interfacial reliability, and output performance.

Many gel-based TENGs still exhibit modest electrical output and gradual performance degradation under long-term cyclic deformation, largely originating from interfacial failure, dehydration, or electrode fatigue. Advanced interfacial engineering strategies, such as chemical bonding, interpenetrating networks, and surface microstructuring, are required to improve electrode–triboelectric layer adhesion and mechanical integrity. Meanwhile, rational matching between gel electrode conductivity and triboelectric friction layer dielectric properties is essential for further improving charge collection efficiency and output stability.

(3) End-of-life sustainability and recyclability.

The end-of-life management of gel-based TENGs is increasingly important, especially for biomedical and wearable applications. Most existing synthetic polymer gels are non-degradable and difficult to recycle. The development of degradable polymer networks, recyclable ionogels, and modular device architectures that enable component separation and reuse represents a promising pathway toward environmentally responsible skin-like electronic systems.

(4) Scalability and system-level integration.

Although high stretchability and self-healing properties have been demonstrated at the laboratory scale, scalable manufacturing and system-level integration remain challenging. Techniques such as 3D printing, spray coating, fiber spinning, weaving, knitting, and roll-to-roll processing offer potential routes for producing large-area and complex-geometry devices. In particular, fiber-shaped and textile-based gel-TENG devices provide unique advantages for large-area coverage and structural conformability, making them highly attractive for full-body robotic skins and wearable perceptual systems. The combination of stretchable conductive gel electrodes with fiber spinning and textile manufacturing technologies enables scalable fabrication of mechanically compliant sensing fabrics. Such architectures offer a practical pathway toward spatially distributed pressure and strain mapping, as well as dynamic force pattern recognition across large surfaces, which are essential for realizing whole-body tactile perception in soft robots and intelligent machines. In parallel, integration of gel-based TENGs with flexible circuits, signal conditioning units, and wireless communication modules will be crucial for enabling real-time data acquisition and practical deployment in wearable electronics, soft robotics, and electronic skin.

Looking forward, continued advances in conductive gel chemistry, interfacial engineering, and device architecture are expected to further promote stretchable conductive gel-based TENGs as a versatile material and device platform for dynamic mechanical signal transduction toward next-generation skin-like electronic systems.

## Figures and Tables

**Table 2:** Comparison of representative self-healing conductive gels for triboelectric applications.

Materials	Types	Self-Healing Principles	Self-Healing Environment	Self-Healing Temperature	Healing Time/Efficiency	Original Mechanical Properties (Stress/Strain)	Refs.
PUA/[EMIM][TFSI]/ACMO	Ionogel	Disulfide bonds/hydrogen bonding	Air (365 nm UV)	RT	10 min/99%	0.29 MPa/546%	[74]
PAAm/[EMIM][TFSI]	Ionogel	Hydrogen bonding/ion–dipole interaction	Water	20 °C	24 h/85%	0.55 MPa/1828%	[75]
PVDF-co-HFP/ZIF-8/[EMIM][TFSI]	Ionogel	Ion-dipole interactions	Air	120 °C	2 min/47.88%	2.14 MPa/1650%	[77]
HEA/LiCl/EG	Deep eutectic gel	Hydrogen bonding	Air	60 °C	24 h/53.57%	0.68 MPa/1400%	[78]
PAA/GA	Hydrogel	Electrostatic interaction	Water	RT	20 min/91%	0.25 MPa/780%	[86]
PAAm/CNT/TA/Ag^+^/gelatin	Hydrogel	Hydrogen bonding	Air	60 °C	2 min/92%	0.4 MPa/280%	[87]
PAA/cellulose/ZnCl_2_	Deep eutectic gel	Metal coordination bonds/hydrogen bonding	Air	RT	1 h/64%	5 MPa/980%	[88]
PVA/Graphene/Chitosan/Agarose/DA·HCl/Na_2_B_4_O_7_	Hydrogel	Hydrogen bonding	Air (NIR)	RT	30 s/94%	0.05 MPa/600%	[89]
PAA/[BMIM]Cl/ChCl	Deep eutectic gel	Hydrogen bonding/ion-dipole interactions	Air (Strong sunlight)	RT	15 min/81.25%	8.8 MPa/1120%	[90]
PAM/OSA/gelatin/Ca^2+^	Hydrogel	Dynamic Schiff base bonds/hydrogen bonding/metal coordination	Air	37 °C	36 h/83.6%	0.630 MPa/2800%	[91]
PAA/SBMA/DA/[BMIM]Cl/solketal	Hydrogel	Electrostatic interactions/hydrogen bonding/hydrophobic association	Air	RT	48 h/94%	0.23 MPa/1400%	[92]
PVA/gelatin/Gly/NaCl/Sodium citrate	Hydrogel	Hydrogen bonding	Air	50 °C	24 h/76.2%	0.34 MPa/420%	[93]
Laponite^®^clay/PEDOT: PSS/OEGMA/DEGMA	Hydrogel	Hydrogen bonding/π–π interactions	Air (75%RH)	25 °C	24 h/95%	0.006 MPa/400%	[94]
TPU/EMI Otf/PCDL-2000/PEG 2000	Ionogel	Ion-dipole interactions/dynamic disulfide/hindered urea bonds	Air	80 °C	2 h/90%	0.5 MPa/990%	[95]

**Table 3:** Representative applications of conductive gel-based triboelectric nanogenerators.

Gel Electrode Composition	TENG Working Mode	Application Examples	Operating Temperature	Additional Features	Operational Lifetime	Durability (Cycles)	Refs.
PAA/Fe_3_O_4_/NH_2_-HBP/[C2mim][EtSO_4_] ionogel	Single-electrode	Motion detection (throat, fingers)	−80 to 250 °C	N.A.	N.A.	5000 cycles	[37]
PAM/gelatin/CNT/TA/Ag^+^/PVP hydrogel	Single-electrode	Motion detection (violation detection in sport events)	−30 °C to RT	Self-healing	N.A.	2000 cycles	[87]
PAA/HPC/Zn^2+^/EG deep eutectic gel	Single-electrode	Motion detection (joint motion, facial expression), gesture recognition	−50 to 80 °C	Anti-humidity	N.A.	14,000 cycles	[110]
PAAm-co-HEA/ZIF-8/LiCl hydrogel	Single-electrode	Motion detection (joint motion), virtual reality gaming	−15 °C to RT	Anti-drying	28 days	50,000 cycles	[159]
PAA/SL/Fe^3+^ deep eutectic gel	Single-electrode	Motion detection (throat, fingers, pulse)	−18 to 60 °C	Anti-drying	N.A.	10,000 cycles	[160]
TA/polypropylene amine/sodium alginate/Mxene hydrogel	Single-electrode	Underwater motion detection (joint motion), encrypted communication	−15 °C to RT	Self-healing	N.A.	5400 cycles	[161]
PVA/NaCl/XG/GL hydrogel	Single-electrode	Motion detection (wrist), underwater robotic grasping detection	RT	N.A.	N.A.	N.A.	[162]
PAM/HPMC/MXene hydrogel	Single-electrode	Handwriting recognition	RT	Anti-drying, anti-humidity	N.A.	5000 cycles	[163]
PAM/SBMA/gelatin/NaCl/Gly hydrogel	Single-electrode	Motion detection (foot), skiing activity monitoring	−20 °C to RT	N.A.	N.A.	6000 cycles	[164]
PVA/NaCl hydrogel	Single-electrode	Motion detection (joint motion, respiration, swallowing)	5 to 45 °C	Anti-humidity	7 days	13,800 cycles	[166]
P(AM-MA)/PEI/β-CD hydrogel	Single-electrode	Gait tactile perception	RT	Self-healing	N.A.	4000 cycles	[167]
PAM/HEMA/LiCl/Laponite XLS hydrogel	Single-electrode	Motion detection (joint motion, respiration), drone haptic control	−23 °C to RT	N.A.	N.A.	4500 cycles	[168]
PVA/CNF/MXene/GL/KOH hydrogel	Single-electrode	Sliding tactile perception (handwriting recognition), intelligent grasping, material recognition	−20 °C to RT	N.A.	N.A.	15,000 cycles	[169]
PVA/BC/[AMIM]Cl ionogel	Single-electrode	Sliding tactile perception (handwriting recognition)	RT	Anti-drying	7 days	10,000 cycles	[170]
PVA/PAM/TA/CNC/SA hydrogel	Single-electrode	Motion detection (joints, throat), handwriting recognition, vehicle pressure detection	−30 to 40 °C	Self-healing	N.A.	6000 cycles	[171]
PAA/[C2mim][EtSO_4_]/Nano ZnO ionogel	Contact separation	Posture detection (vehicle/ship warning)	RT	N.A.	N.A.	8000 cycles	[172]
PAAm/clay/KI/Gly hydrogel	Single-electrode	Braille tactile recognition	−10 to 80 °C	Self-healing	N.A.	3000 cycles	[173]

## Data Availability

No data was used for the research described in the article.

## Abbreviations

The following abbreviations are used in this manuscript:

**Abbreviation**	**Full Name**
[AMIM]Cl	1-allyl-3-methylimidazolium chloride
[BMIm]Zn_x_Cl_y_	a metal halide ionic liquid
[BMIM]Cl	1-butyl-3-methylimidazolium chloride
[C2mim][EtSO_4_]	1-ethyl-3-methylimidazolium ethyl sulfate
[EMIM][TFSI]	1-ethyl-3-methylimidazolium bis(trifluoromethyl sulfonyl) imide
[VBIM][BF_4_]	1-vinyl-3-butylimidazolium tetrafluoroborate
[VBIM]Br	1-vinyl-3-butylimidazolium bromide
1,2-HeD	1,2-hexanediol
AA	acrylic acid
AAm	acrylamide
AC	allyl chloride
ACMO	4-acryloylmorpholine
AMPS	2-acrylamido-2-methyl-1-propanesulfonic acid
APS	ammonium persulphate
BA	betaine
BC	bacterial cellulose
BWT	black wattle bark tannin
CA	carboxymethyl chitosan
CB-OH	2-((2-hydroxy-3-(methacryloyloxy) propyl) dimethyl ammonio) acetate
ChCl	choline chloride
CNC	cellulose nanocrystal
CNFs	cellulose nanofibers
CNTs	carbon nanotubes
CoN CNT	cobalt nanoparticle encapsulated nitrogen-doped carbon nanotubes
CS	chitosan
DA	Dodecyl Acrylate
DA·HCl	dopamine hydrochloric acid
DEGMA	di(ethylene glycol) methyl ether methacrylate
DMAPS	3-dimethyl (methacryloyloxyethyl) ammonium propane sulfonate
EG	ethylene glycol
EMI Otf	1-ethyl-3-methylimidazolium trifluoromethanesulfonate
FeCl_3_·6H_2_O	ferric chloride hexahydrate
GA	gum arabic
GL	glycerin
Gly	glycerol
GMA	glycidyl methacrylate
GO	graphene oxide
HEA	N-hydroxyethyl acrylamide
HEMA	2-hydroxyethyl methacrylate
HFP	hexafluoropropylene
HPC	hydroxypropyl cellulose
HPMC	hydroxypropyl methylcellulose
IA	itaconic acid
KI	potassium iodide
KOH	potassium hydroxide
LA	lactic acid
LiCl	lithium chloride
MA	maleic acid
MAM	methacrylamide
Na_2_B_4_O_7_	Sodium Tetraborate
NaCl	sodium chloride
NaClO_4_	sodium perchlorate
NH_2_-HBP	amino terminated hyperbranched polyamide
NVP	N-vinylpyrrolidone
OEGMA	oligo(ethylene glycol) methyl ether methacrylate
OSA	oxidized sodium alginate
P(AM-MA)	poly(acrylamide-co-maleic acid)
PA	phytic acid
PAA	poly(acrylic acid)
PAAm or PAM	poly(acrylamide)
PC	propylene carbonate
PCDL-2000	polycarbonate Diol-2000
PDA	polydopamine
PEDOT:PSS	poly(3,4-ethylene dioxythiophene): poly(styrene sulfonate)
PEG 200	polyethylene glycol 200
PEG 2000	polyethylene glycol 2000
PEGDA 1000	poly(ethylene glycol)diacrylate
PEI	polyethylenimide
PG	propylene glycol
PHFP	poly(hexafluoropropylene)
PUA	poly(urethane acrylate)
PVA	poly(vinyl alcohol)
PVDF	poly(vinylidene fluoride)
PVDF-co-HFP	poly(vinylidene fluoride-co-hexafluoropropylene)
PVP	poly(vinylpyrrolidone)
RT	room temperature
SBMA	N-(3-sulfopropyl)-N-(methacryloxyethyl)-N,N-dimethylammonium betaine
SL	sodium lignosulfonate
solketal	2,2-dimethyl-1,3-dioxolane-4-methanol
TA	tannic acid
TFEA	2,2,2-trifluoroethyl acrylate
TOCNFs	2,2,6,6-tetramethylpiperidine-1-oxyl radical oxidized cellulose nanofibers
TPU	thermoplastic polyurethane
Tr	trehalose
VMT	vermiculite
WPU	waterborne polyurethanes
XG	xanthan gum
ZIF-8	zeolitic imidazolate framework-8
β-CD	β-cyclodextrin
